# The modular architecture of sigma factors in cyanobacteria: a framework to assess their diversity and understand their evolution

**DOI:** 10.1186/s12864-024-10415-x

**Published:** 2024-05-24

**Authors:** Marine Gevin, Amel Latifi, Emmanuel Talla

**Affiliations:** Aix Marseille Univ, CNRS, Laboratoire de Chimie Bactérienne, LCB, IMM, Marseille, France

**Keywords:** Cyanobacteria, Gene transcription, Modular domains, Sigma factors

## Abstract

**Background:**

Bacterial RNA polymerase holoenzyme requires sigma70 factors to start transcription by identifying promoter elements. Cyanobacteria possess multiple sigma70 factors to adapt to a wide variety of ecological niches. These factors are grouped into two categories: primary sigma factor initiates transcription of housekeeping genes during normal growth conditions, while alternative sigma factors initiate transcription of specific genes under particular conditions. However, the present classification does not consider the modular organization of their structural domains, introducing therefore multiple functional and structural biases. A comprehensive analysis of this protein family in cyanobacteria is needed to address these limitations.

**Results:**

We investigated the structure and evolution of sigma70 factors in cyanobacteria, analyzing their modular architecture and variation among unicellular, filamentous, and heterocyst-forming morphotypes. 4,193 sigma70 homologs were found with 59 distinct modular patterns, including six essential and 29 accessory domains, such as DUF6596. 90% of cyanobacteria typically have 5 to 17 sigma70 homologs and this number likely depends on the strain morphotype, the taxonomic order and the genome size. We classified sigma70 factors into 12 clans and 36 families. According to taxonomic orders and phenotypic traits, the number of homologs within the 14 main families was variable, with the A.1 family including the primary sigma factor since this family was found in all cyanobacterial species. The A.1, A.5, C.1, E.1, J.1, and K.1 families were found to be key sigma families that distinguish heterocyst-forming strains. To explain the diversification and evolution of sigma70, we propose an evolutionary scenario rooted in the diversification of a common ancestor of the A1 family. This scenario is characterized by evolutionary events including domain losses, gains, insertions, and modifications. The high occurrence of the DUF6596 domain in bacterial sigma70 proteins, and its association with the highest prevalence observed in Actinobacteria, suggests that this domain might be important for sigma70 function. It also implies that the domain could have emerged in Actinobacteria and been transferred through horizontal gene transfer.

**Conclusion:**

Our analysis provides detailed insights into the modular domain architecture of sigma70, introducing a novel robust classification. It also proposes an evolutionary scenario explaining their diversity across different taxonomical orders.

**Supplementary Information:**

The online version contains supplementary material available at 10.1186/s12864-024-10415-x.

## Introduction

 Cyanobacteria are gram-negative prokaryotes that perform oxygenic photosynthesis or have lost this ability over time [1]. They are also the simplest organisms yet shown to exhibit circadian rhythms of biological activities under the control of a well-known endogenous clock [2]. The rise of cyanobacteria is thought to have played a major role in our planet’s evolution towards oxygen accumulation in the atmosphere and water [3]. Besides O_2_ production, cyanobacteria act as major carbon sinks as they fix atmospheric carbon dioxide (CO_2_). Cyanobacteria also play a crucial role in the global nitrogen cycle [4]; many of them can reduce atmospheric nitrogen (N_2_), making it bioavailable for heterotrophic organisms to assimilate [5]. Indeed, filamentous Nostoc strains fix atmospheric nitrogen aerobically in complex, specialized cells called heterocysts, whereas other filamentous and some unicellular strains are capable of fixing nitrogen under anoxic conditions [4]. The versatile metabolism of cyanobacteria explains the great interest they attract in agriculture, aquatic ecology, and environmental protection fields. Consequently, the successful use of these bacteria in a wide range of biotechnologies is well-established nowadays. This includes synthesizing high-value secondary metabolites and biologically active compounds, biofuel production, and bioremediation [6–8].

 Cyanobacteria colonized almost all aquatic and terrestrial ecosystems [9]. In addition to this broad ecological distribution, they display a wide morphologic, and genetic diversity [10]. Based on morphology mode, cyanobacteria can be subdivided into three main groups: unicellular, filamentous, and filamentous capable of cell differentiation. These groups were classified into 5 mono and polyphyletic sub-groups or sections [11, 12]. Sections I and II gather unicellular species, subdivided according to their division mode. Sections III to V gather multicellular species organized in filaments or trichomes: section III species do not differentiate into heterocysts whereas sections IV and V species do. In addition, members in section V present branched filaments.

Cyanobacteria can perceive and respond to a multitude of environmental signals and tightly synchronize their metabolic and cellular processes to their niches. As in many other organisms, the regulation of gene expression in cyanobacteria plays a major role in modulating biological activities and in shaping adaptive responses. In this respect, programming the RNA polymerase (RNAP) catalytic core enzyme with the sigma70 subunit that gives its ability to recognize and interact with target promoters is a key step in gene transcription regulation. Typically, transcription of essential, housekeeping genes is ensured by RNAP carrying the primary sigma factor, and control of gene transcription in response to environmental and/or physiological stimuli requires alternative sigma70 factors. While two distinct classes, sigma70 and sigma54 have been identified in eubacteria, only sigma70 encoding genes are present in cyanobacterial genomes analyzed so far [13, 14].

**Fig. 1:**
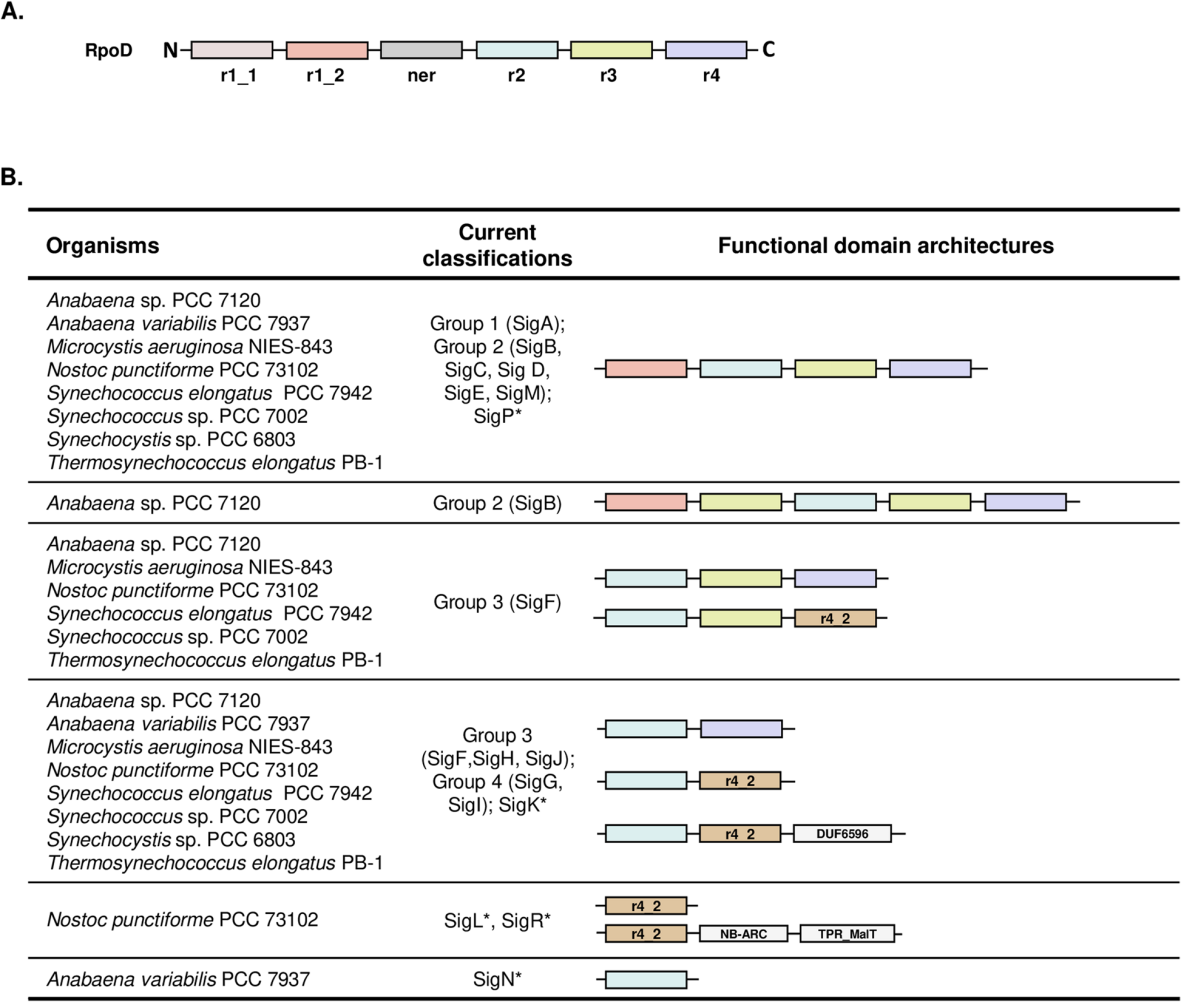
**A** Structural domain organization of the* E. coli *canonical sigma70. The protein sequence has been divided into 6 functional domains based on sequence conservation with domain profiles of the Pfam database. **B** Protein domain patterns with current classification of known and representative sigma proteins in cyanobacteria. The standard RpoD protein of *Escherichia coli* K12 is also shown. Domains are shown with distinct color boxes. *, stands for unclassified sigma factors. Domain architectures of each group of representative sigma70 proteins are presented as revealed by our analysis. Domain abbreviations are: r1_1 for Sigma70_r1_1; r1_2, Sigma70_r1_2; ner, Sigma70_ner; r2, Sigma70_r2; r3, Sigma70_r3; r4, Sigma70_r4; and r4_2, Sigma70_r4_2. The description of domains is shown in Additional file 1: Tables S2 & S3. Structurally similar domains are indicated by the same colors as in panel A

The canonical sigma70 proteins reveal 4 functional and conserved domains (Fig. 1A) [13, 14] Domains r2 and r4 are well conserved in all members of the sigma70 family and are involved in binding to RNA polymerase and in promoter recognition and melting. The recognition of the − 35 promoter element is mediated by a helix-turn-helix unit in domain r4 and the amino acid residues important for both the − 10 region recognition and promoter melting are located on α-helix region r2 [15, 16]. Based on domain-composition and phylogeny relationships, the sigma70 family was subdivided into four groups [13]. The primary sigma70 factors compose the group 1. They all carry at least four structural and essential domains including r1_2, r2, r3, and r4 (or r4_2, a variation of r4 domain) along with their sub-domains, and r1_1 and ner domains in particular cases such as in *E. coli*. Alternative sigma70 factors differ from primary factors by lacking r1_1 and ner and having variable domains. Group 2 sigma70 factors are similar in sequence to the primary sigma70 factors but are not essential to growth. Group 3 sigma70 factors are less similar to those of Group 1. They include proteins required for heat-shock response, motility, and sporulation [13, 17, 18]. Members of Group 4 have the most distant sequence similarity with other sigma70 factors. They include those known as extracellular sigma70 factors because they regulate cellular functions related to transport or cell surface [15, 19].

The current classification of sigma70 factors in cyanobacteria is broadly based on the four groups described above. The classification of sigma70 factors was initially based on *Synechocystis* PCC 6803 proteins [20, 21]. Later, it was expanded to 6 model organisms, and then to 8 based on phylogenetic determinants [16, 21].

However, this classification was established by studying only a limited number of cyanobacterial genomes, so our knowledge of the diversity of these factors at a phylum-wide level is still limited. In addition, it should be noted that sigma70 proteins are made up of various domains, yet their modularity has not been fully considered.

In this study, we analyzed the modular architecture distribution of sigma70 factors in cyanobacteria, utilizing a large genomic scale approach. Based on the results obtained, it is concluded that this approach is a reliable tool for classification purposes. Furthermore, we used the identification and classification of sigma70 proteins to investigate their distribution among different cyanobacterial morphotypes. Finally, we propose an evolutionary hypothesis that explains how the modular structure of these proteins could have led to their diversification and framed their role in regulating gene expression.

## Results

### Identifying the domain architectures of representative sigma70 factors in cyanobacteria

To analyze the domain structure of sigma70 factors within the cyanobacterial phylum, we first established the architecture of representative factors, which have been used in prior classification studies [16, 21]. In the first step, we identified all the domains that compose these factors, covering proteins from eight distinct species: *Anabaena* sp. PCC 7120, *Anabaena variabilis* PCC 7937, *Microcystis aeruginosa* NIES-843, *Nostoc punctiforme* PCC 73102, *Synechococcus* sp. PCC 7002, *Synechococcus elongatus* PCC 7942, *Synechocystis* PCC 6803, and *Thermosynechococcus vestitus* BP-1 [16, 21]. These representative proteins (62 in total) were previously grouped into 17 families (named SigA to SigR) (Additional file 1: Table S1). Sigma proteins from *E. coli* K12 (from sigma19 to sigma70) were also included in the representative protein set as they were used as seeds in the classification studies of sigma70 factors in cyanobacteria [16, 21]. We observed the presence of 6 essential domains (Sigma70_r1_2 [r1_2], Sigma70_r2 [r2], Sigma70_r3 [r3], Sigma70_r4 [r4], Sigma70_r4_2 [r4_2], and Sigma70_ECF [ECF, for Extracytoplasmic function sigma factor]) and 3 additional domains (DUF6596, NB-ARC, and TPR-MalT) for SigA to SigR in cyanobacterial proteins. DUF6596 is a domain of unknown function while the NB-ARC domain is a functional ATPase domain shared by plant disease proteins and regulators of cell death in animals [22, 23]. The TPR_MalT domain contains a series of tetracopeptide repeats (TPR) found in the transcriptional regulator MalT and related proteins [24].

The analysis of the domain architectures of the representative proteins indicated the presence of 10 distinct modular domain organizations: (*i*) r1_2*r2*r3*r4 pattern composed of four main types of domains and for which the representative proteins have been classified in SigA, SigB, SigC, SigE, SigM and SigP; (*ii*) r1_2*r3*r2*r3*r4 pattern currently classified as SigB; (*iii*) r2*r3*r4 and r2*r3*r4_2 patterns (currently classified as SigF); (*iv*) r2*r4, r2*r4_2 and r2*r4_2*DUF6596 patterns (currently classified as SigF, SigG, SigH, SigI, SigJ, and SigK); (*v*) r4_2 et r4_2*NB-ARC*TPR_MalT patterns (classified as SigL and SigR, respectively) and (*vi*) r2 pattern classified as SigN (Fig. 1B and Additional file 1: Table S1).

Based on the collected data for representative sigma70 proteins, it can be inferred that (*i*) all of the main and functional domains in RpoD of *E. coli* were located within the representative seed sigma70 proteins, except for r1_1 and ner domains). The non-identification of such r1_1 and ner domains could be because the two domain profiles are not appropriate for cyanobacteria due to the taxonomic distance, or that these domains are truly absent. (*ii*) Other non-canonical functional domains such as DUF6596, NB-ARC, and TPR_MalT are associated with sigma70 proteins and therefore could have a functional impact on their function; (*iii*) consequently, the functional domains associated with sigma70 can be divided into two categories: essential (r1_2, r2, r3, r4, r4_2 and ECF) and accessory domains (r1_1, ner, DUF6596, NB-ARC et TPR_MalT) (Additional file 1: Table S2).

Interestingly, the analysis of domain architecture among sigma70 representative proteins revealed that the modular organization does not match the distribution of these proteins in the 10 patterns mentioned above, indicating strong biases in the current classification. For instance, the same domain architecture was associated with distinct groups of proteins (e.g., r1.2*r2*r3*r4 pattern currently classified as SigA, SigB, SigC, SigD, SigE, SigM or SigP) (Fig. 1B). Conversely, members of the same family display different domain architectures (e.g., SigB and SigF). Furthermore, proteins with the same pattern of essential domains may or may not have additional domains (e.g., SigK and SigL, with or without DUF6596) (Fig. 1B and Additional file 1: TableS1).

### Deciphering the global distribution of sigma70 factors in cyanobacteria

With the aim to improve sigma70 factors classification, 361 genomes of cyanobacteria covering the 3 cyanobacterial morphotypes (unicellular, filamentous, and heterocyst-forming strains) were analyzed for the presence of sigma70 proteins and the organization of their structural domains (see Materials and Methods). As expected, given the function ensured by these factors, sigma70 homologs were detected in all the analyzed genomes. In total, we identified 4193 sigma70 homologs which represent 0.25% of overall analyzed proteins (Additional file 2: Table S1). The data revealed 59 distinct modular organizations, including the essential domains described above (r1.2, r2, r3, r4, r4_2, and ECF) and 29 distinct accessory domains (Additional file 1: TableS3). Surprisingly, accessory domains were located exclusively at N- or C-terminal extremities of the proteins (i.e., never between essential domains). The most represented accessory domains were: DUF6596 (40.56%), NB-ARC (9.09%), WD40 (8.39%), zf-C4_ClpX (6.29%), Ank_2 (5.59%), TPR_MalT (4.20%), AAA_35 (2.80%), CRISPR_Cas6, and CRIPR_Cas6_N (2.10%). The DUF6596, NB-ARC, and TPR_MalT were presented above, and the predicted functions of other accessory domains are summarized in Additional file 1: Table S3.

The number of sigma70-encoding genes among the 361 studied genomes was found to be highly variable with a minimum of 3 homologs (in Candidatus *Atelocyanobacterium thalassa*) and a maximum of 37 homologs (in *Leptolyngbya valderiana*,). The data also showed that 90% of the organisms had 5 to 17 homologs with an average of 12 homologs per genome (Additional file 3: Fig. S1 & S2). Interestingly, the distribution of homologs according to the strain morphotype demonstrated that the number of homologs is statistically higher in multicellular heterocyst-forming organisms (H, average of 13.8 homologs per genome), compared to the other 2 morphotypes (U and F, average of 11.01 homologs per genome) (Additional file 3: Fig. S2). The possibility that the number of sigma 70 homologs may vary as a consequence of genome size was first analyzed by examining the distribution of genome sizes (defined here as the total number of coding genes in the genome) across different strain morphotypes. The analysis revealed that the genome sizes are notably larger in H-type organisms compared to F and U types (Additional file 3: Fig. S3). Subsequently, the relationship between the number of sigma70 homologs and the total protein-coding genes (CDS) in the genome was plotted, as shown in Additional file 3: Fig. S4. The data illustrate that the number of sigma70 homologs tends to increase with genome size across the different cyanobacterial morphotypes. Of note, for the genome sizes between 3000 and 7000 CDS, there is no significant variation of the number of sigma homologs between U, F and H-type organisms, although there are exceptions. For example, despite having similar genome sizes of approximately 6000 CDS, *Acaryochloris marina* S15 (U-type) has 33 homologs, while *Trichormus variabilis* NIES-23 (H-type) and *Microcoleus asticus* IPMA8 (F-type) have only 18 and 12 sigma homologs, respectively. For genome sizes over 7000 CDS, the number of sigma homologs is higher in H-type organisms, probably due to the increasing sizes of their genomes during the evolution.

To explore further, the distribution of sigma70 factors was analyzed at the level of taxonomic orders, which were grouped based on their phenotypic features.

**Fig. 2:**
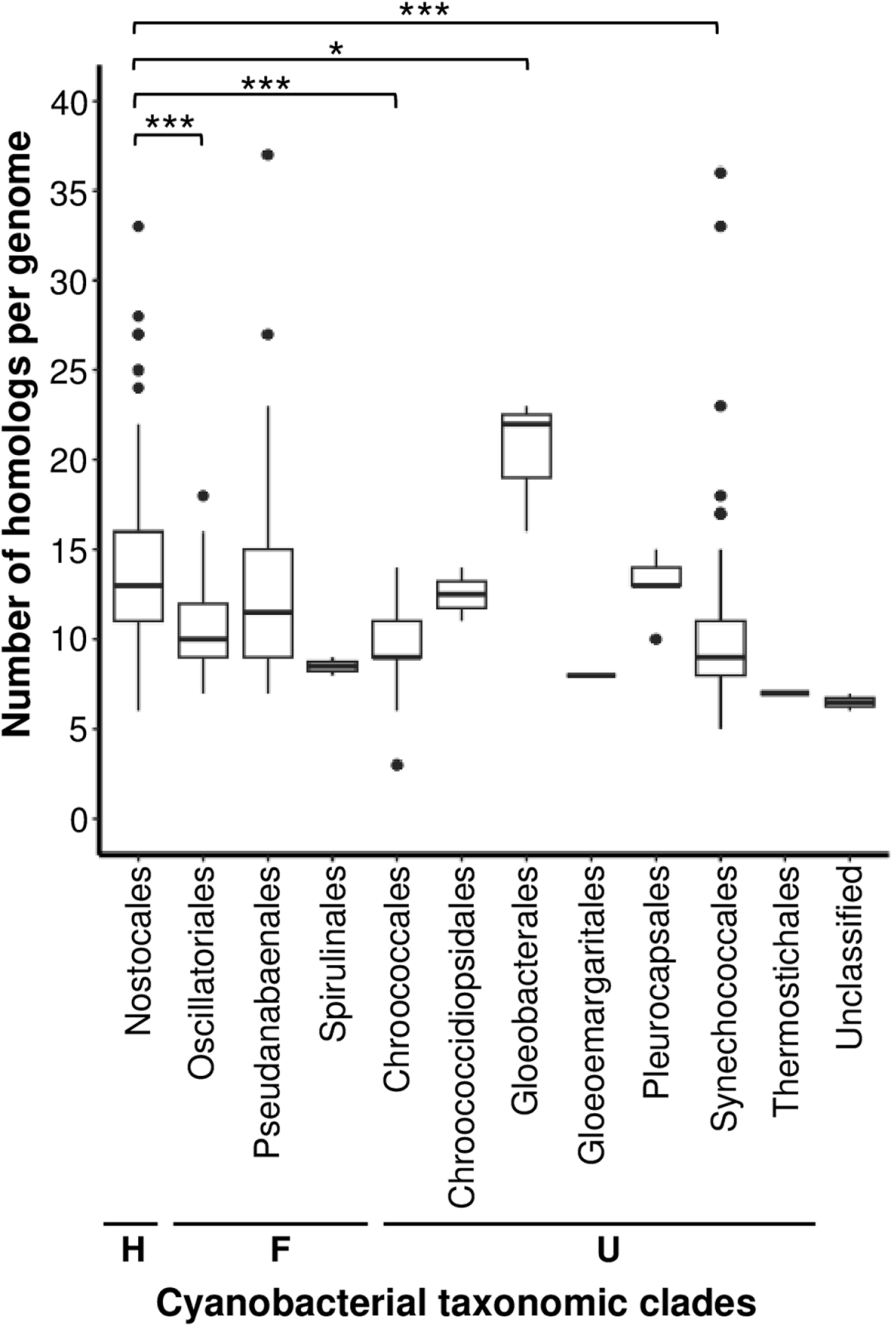
Distribution of the number of sigma70 homologs per genome within the cyanobacterial taxonomic orders. Comparisons are relative to Nostocales order, with * or *** when the statistical significance (Wilcoxon-Mann-Whitney tests, *p*-value) was below 0.05 or 0.001, respectively. H, heterocyst-forming; F, filamentous non-heterocyst; and U, unicellular organisms

The data presented in Fig. 2 indicate that the distribution of homologs per genome according to the taxonomic orders displayed considerably higher amounts of sigma70 in Nostocales (H-type strains), with an average of 13.8 sigma70 proteins, compared to Oscillatoriales (F-type strains), with an average of 10.46, or Chroococcales and Synechococcales (both U-type strains), with averages of 9.66 and 9.79, respectively. Concurrent with observations at the morphotype level, it can be concluded that the variation in the number of homologs among cyanobacterial strains likely reflects a combination of the strain morphotype, the taxonomic order and the genome size.

### Modularity-based classification of sigma70 factors in cyanobacteria

**Fig. 3:**
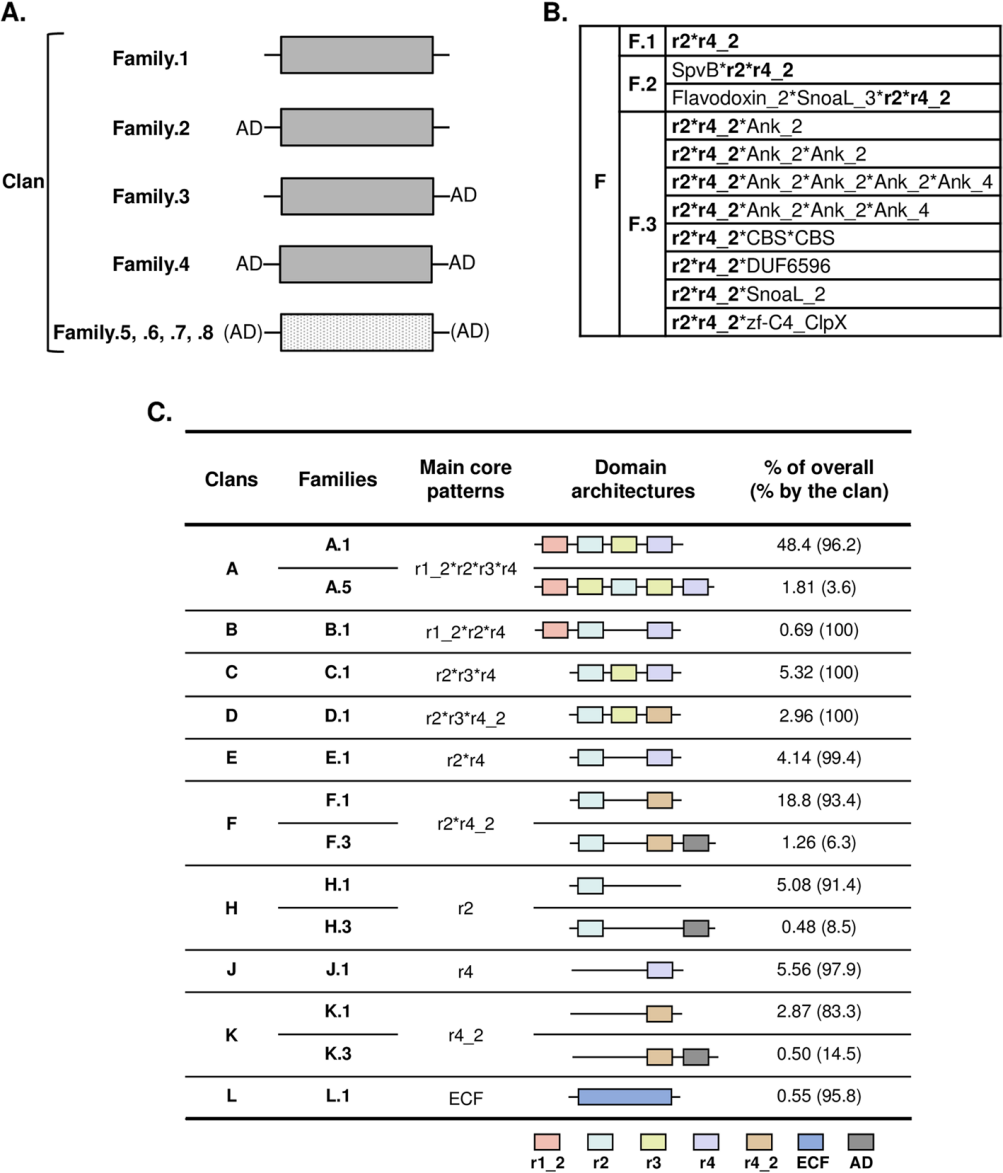
**A** Classification model of the sigma70 homologs into clans and protein families. Canonical and non-canonical core pattern of domains are shown in plain and dotted grey boxes, respectively. Inside a clan, a group of protein (here a family) with a canonical core pattern is numbered 1, 2, 3, or 4 depending of the presence or absence of the accessory domain (AD). Families with non-canonical core patterns are numbered 5,6,7, or 8. See text for details.  **B** Representation of the clan F as an example of clans with families . Core patterns are in bold. AD is in standard characters, and their functional descriptions are in Additional file 1: Tables S2 & S3. **C** Distribution of Sigma proteins in cyanobacteria according to our novel classification model. Main clans and families are shown with their core patterns, domain architectures, and proportions (in percentage) over the total genome and by the clan. Details are shown in Additional file 1: Table S4. Domains are shown with distinct color boxes. Domain abbreviations are: r1_2, Sigma70_r1_2; r2, Sigma70_r2; r3, Sigma70_r3; r4, Sigma70_r4; r4_2, Sigma70_r4_2; and ECF, Sigma70_ECF. The description of domains is displayed in Additional file 1: Tables S2 & S3

Given that the modularity of proteins may infer functional plasticity and specificity, it is possible to identify domain organization as a characteristic of a group of proteins that belong to a specific functional family. Therefore, we used the modular structures of the domains presented above as a tool for the classification of sigma70 factors. Our methodology was set up as follows (Fig. 3A): (*i*) families and clans (groups of families) were used to build a hierarchical classification based on the organization of essential domains and on the presence/absence of accessory domains; (*ii*) a clan was defined as a set of sigma70 homologs sharing the same set of essential domains (e.g., clan A with the four essential domains r1_2, r2, r3, and r4; and clan F with two essential domains r2 and r4_2) and identical architecture of essential domains defined the core patterns of domains. The core pattern of domains could be canonical (which referred to the domain architecture with a single copy of each seed essential domain) or non-canonical (for which the core pattern harbors duplicated essential domains); (iii) within the same clan, a family is defined as a cluster of homologs that exhibits the same core pattern of domains (i.e., identical domain architecture). Families are distinguished by the presence or not of an accessory domain (AD). Inside a clan, a group of protein with a canonical core pattern but without AD are numbered 1. The ones with AD at the N-terminal or C-terminal region of the core patterns are numbered 2 or 3, respectively. Cluster of homologs with canonical core pattern and AD on both side of the protein are numbered 4. A given example of the clan F with 3 families are shown in Fig. 3B). Similarly, families with non-canonical core patterns (e.g., r1_2*r2*r3*r4) are numbered 5,6,7 and 8 (Fig. 3A). Inside a clan, family numbers can be extended to 9, 10, 11, 12, … in case new sigma70 homologs with distinct non-canonical core patterns come to be identified.

The obtained results revealed that the sigma70 factors can be classified into 12 primary clans (A-L) with defined core patterns and a miscellaneous clan named Z with undetermined core patterns (Additional file 1: Table S4). The 4193 obtained homologs were then subgrouped into 36 families. Figure 3C shows the distribution of the 10 major clans and their respective families. The representations of the members of each family within the genomes and the clan are given (in percentage). Clan A was the largest clan as it gathered the essential sigma70 factors as described in Fig. 1A. As observed for representative sigma70 proteins (Fig. 1B), when only one functional domain was present in the protein, it was either r2, r4 (or r4_2). Moreover, these two domains (r2 and r4) were present in all families, the only exception being the clan L (family L.1) (Fig. 3C). This observation is consistent with the essentiality of these 2 domains for interaction with promoters (see Introduction section). Whether members of the L clan can function as sigma70 subunits remains to be elucidated.

We utilized the classification of sigma70 factors into clans and families to propose a new convention name for each of the 4193 sigma70 factor units. To achieve this, we combined suffixes (e.g., digits 0.1, 0.2, 0.3, etc…) with the family name to achieve this. This resulted in a novel name associated with each sigma factor unit, such as SigA.1.1, SigA.1.2, and SigA.1.3, in each genome. If a sigma70 family contained multiple homologs within a genome, the digit numbers of the sigma70 novel names were arbitrarily set among the homologs. We followed this procedure for all sigma70 homologs in cyanobacteria (Additional file 2: Table S1), and the novel sigma names of cyanobacteria model organisms were presented in Table 1. This standardized nomenclature can be used for sigma70 genes obtained in future genome sequencing projects which will prevent the introduction of biases. A further advantage of this nomenclature is that it can be applied to all prokaryotic genomes, homogenizing current disparate and biased nomenclatures.

**Table 1:** Novel names of sigma70 proteins in cyanobacteria model organisms, according to our classification. Abbreviations of the cyanobacteria model organisms are: *NIES-843*, *Microcystis aeruginosa* NIES-843; PCC 73102, *Nostoc punctiforme* PCC 73102; PCC 7120, *Nostoc* sp. PCC 7120; PCC 7942, *Synechococcus elongatus* PCC 7942; PCC 7002, Synechococcus sp. PCC 7002; PCC 6803, *Synechocystis* sp. PCC 6803; BP-1, *Thermosynechococcus vestitus* BP-1; ATCC 29413, *Trichormus variabilis* ATCC 29413. nc, non-classified sigma70 factors

		Proposal names of sigma protein units in cyanobacteria model organisms							
Current classifications and denominations of sigma factor units		NIES-843	PCC 73102	PCC 7120	PCC 7942	PCC 7002	PCC 6803	BP-1	ATCC 29413
***Group 1***	***SigA***	SigA.1.1	-	SigA.1.1	SigA.1.1	SigA.1.1	SigA.1.1	SigA.1.1	-
***Group 2***	***SigB***	SigA.1.2	-	SigA.1.2->4 SigA.5.1	SigA.1.2	SigA.1.2	SigA.1.2	SigA.1.2	-
	***SigC***	SigA.1.3	SigA.1.1	SigA.1.5	SigA.1.3	SigA.1.3	SigA.1.3	SigA.1.3	-
	***SigD***	SigA.1.4	-	SigA.1.6	SigA.1.4	SigA.1.4	SigA.1.4	SigA.1.4	-
	***SigE***	SigA.1.5	-	SigA.1.7	-	SigA.1.5	SigA.1.5	-	-
	***SigM***	-	-	-	SigA.1.5->6	-	-	-	-
***Group 3***	***SigF***	SigD.1.1	SigC.1.1	SigC.1.1	SigC.1.1	SigC.1.1	SigE.1.1	SigD.1.1	-
	***SigH***	-	-	-	-	SigF.1.2	SigE.1.2	SigF.1.2	-
	***SigJ***	-	SigF.1.2	SigF.1.3	SigF.1.2	-	-	-	-
***Group 4***	***SigG***	SigF.1.1	SigF.1.1	SigF.1.1	SigF.1.1	SigF.1.1	SigF.1.1	SigF.1.1	-
	***SigI***	-		SigF.1.2	SigE.1.1	-	SigE.1.3	-	-
***Unclassified group***	***SigK***	-	SigF.3.1	-	-	-	-	-	-
	***SigL***	-	SigK.3.1	-	-	-	-	-	-
	***SigN***	-	-	-	-	-	-	-	SigH.1.1
	***SigP***	-	-	-	-	-	-	-	SigA.1.1
	***SigR***	-	SigK.1.1	-	-	-	-	-	-
	***nc***	SigF.1.2 SigJ.1.1	SigA.1.2->9 SigH.1.1 SigJ.1.1->2 SigZ.5.1	SigJ.1.1->3	-	-	-	-	SigA.1.2->6 SigA.5 0.1 SigC.1.1 SigD.1.1 SigF.1.1 SigJ.1.1->2

Correspondances between proposal names of sigma factor units and Uniprot/Refseq accession numbers or gene locus are in Additional Table 2: Table S1. In the case of Nostoc sp. PCC 7120, here are the novel names of sigma factor units with their gene locus tags in parenthesis: SigA.1.1(all5263), SigA.1.2(all7615), SigA.1.3(all7608), SigA.1.4(alr3800), SigA.1.5(all1692), SigA.1.6(alr3810), SigA.1.7(alr4249), SigA.5.1(all7179), SigC.1.1(all3853), SigF.1.1(alr0277), SigF.1.2(alr3280), SigF.1.3(all2193), SigJ.1.1(alr7311), SigJ.1.2(alr1376) and SigJ.1.3(all3581)

### Distribution of major sigma70 families in the cyanobacterial phylum

**Fig. 4:**
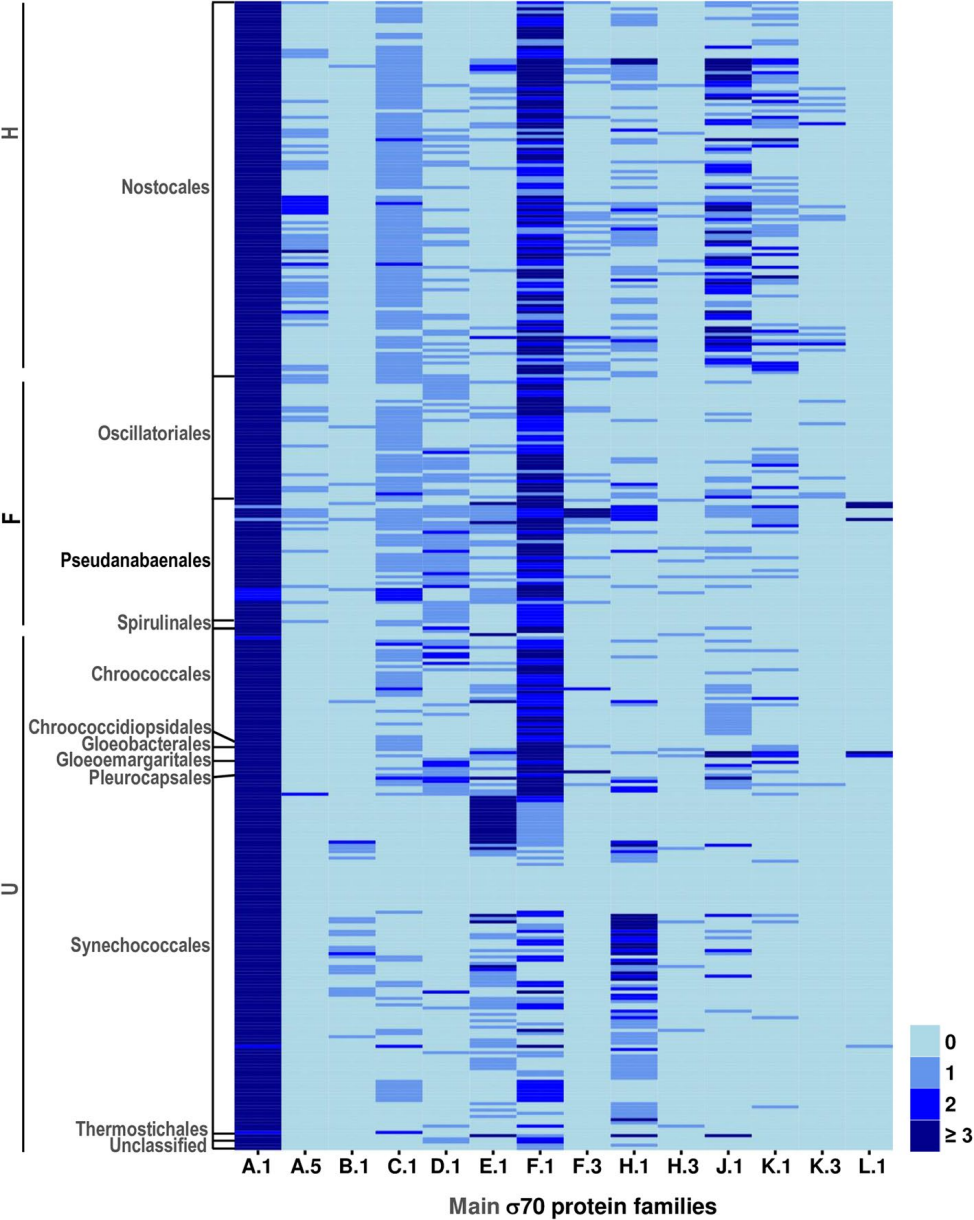
Heatmap graphical representation of the number of homologs within organisms. Main sigma70 protein families are shown with their associated taxonomic orders and the phenotypic traits (H, heterocyst-forming; F, filamentous non-heterocystous; and U, unicellular organisms). Each line corresponds to one cyanobacterial strain. The color scale (0, 1, 2, or > = 3) represents the number of homologs in each genome

To investigate whether the distribution of sigma70 genes differs based on the morphotype of strains or their phylogenetic position within the phylum, we examined the correlation between the number of homologs in the 14 primary families, classified by taxonomic orders and phenotypic traits (Fig. 4 and Additional file 2: Tables S2 & S3). The data presented in the heatmap of Fig. 4 showed that the distribution of homologs within the main families analyzed was highly variable. Members of the A.1 family were found in at least one copy (with a maximum of 19 homologs) in all organisms. The abundant presence of members in this family implies that this sigma70 factor existed in the common ancestor of cyanobacteria. Consequently, certain members continued to serve as the primary sigma70 factor, retaining the four crucial domains (namely: r1_2, r2, r3, and r4/r4_2). The F.1 (r2*r4_2) family had a broader distribution than other families and was found in all organisms but with lower copy numbers in F.1 Synechococcales compared to A.1 members. Members from the families A.5 (r1.2*r3*r2*r3*r4), C.1 (r2*r3*r4), J.1 (r4), and K.1 (r4_2) were notably prevalent within the Nostocales (H-type) compared to Synechoccocales (U-type) groups. The opposite observation was drawn for E.1 (r2*r4) and H.1 (r2) family members. In contrast, homologs belonging to families F.3 (r2*r4_2*AD) and K.3 (r4_2*AD) were primarily observed in the Nostocales, Oscillatoriales, and Pseudanabaenales groups. Meanwhile, the B.1 family (r1_2*r2*r4) appeared to be particularly abundant in the Synechoccocales group. Intriguingly, the L.1 family, characterized solely by an ECF domain, was absent in heterocyst-forming cyanobacteria.

Interestingly, 14 species of Prochlorococcus, along with the *Synechococcus* sp. WH 8119 strain -both belonging to the order Synechococcales - exclusively contained homologs from the A.1 family (Additional file 2: Table S2). Conversely, none of the strains possessed members from all of the major 14 families. A notable near-exception is the genome of *Leptolyngbya boryana* strains, which exhibited homologs from 10 out of the 14 principal sigma70 families (Fig. 4 and Additional file 2: Tables S2).

**Fig. 5:**
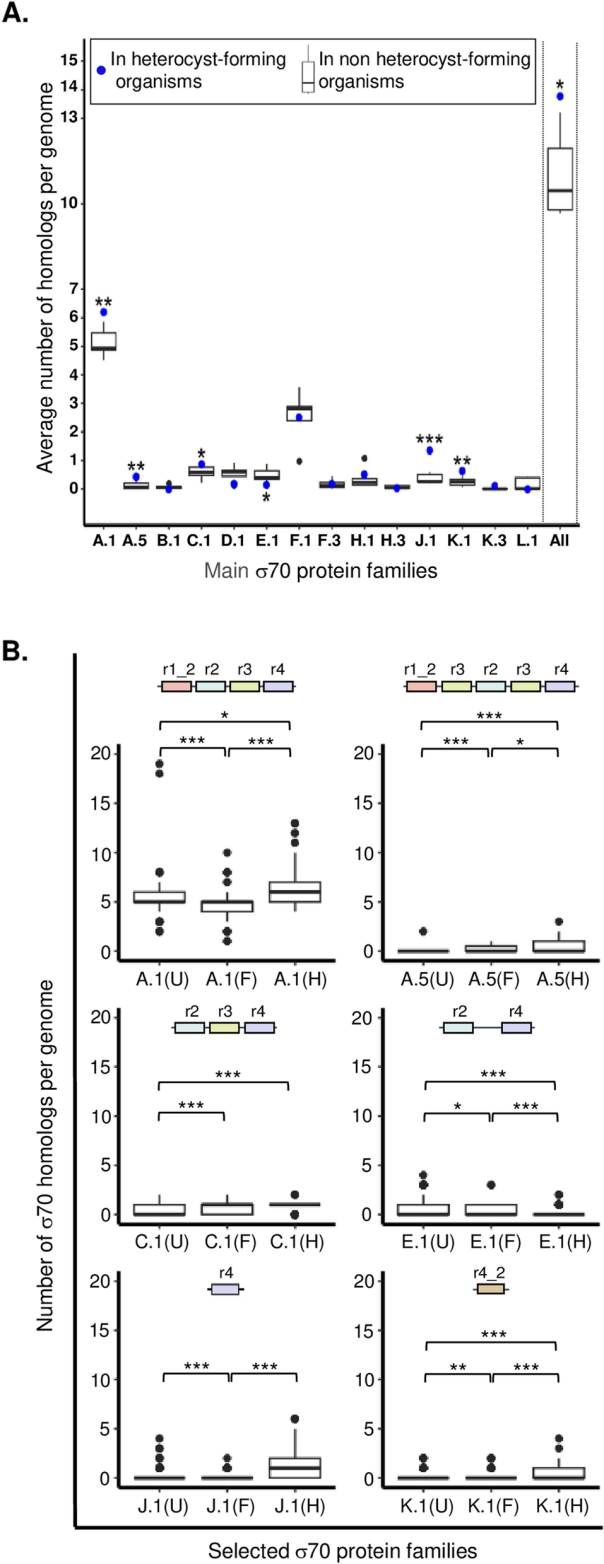
**A** Boxplot showing the average number of homolog genes per genome within the morphotype groups. For each main protein family, blue dots are related to heterocyst-forming organisms (here the average number of homologs in Nostocales), with *, ** or *** on the top [if the number of homologs is significantly higher in heterocyst-forming organisms vs. non heterocysts-forming (including unicellular, filamentous and unclassified) organisms] or on the bottom [for the opposite] when the statistical significance (one-sample t-test, p-value) was below 0.05, 0.01 or 0.001, respectively. “All” refers to the sum of the overall data. **B** Distribution of the number of homologs per genome in function of morphological and physiological traits. The six protein families displaying significant biases between heterocyst-forming and non-heterocystous organisms in panel A are shown and their domain organizations indicated. The information related to the other families is given in Additional file 3: Fig. S5. For each protein family, all possible statistical comparisons were performed between unicellular (U), non-heterocyst-forming (F) and heterocyst-forming (H) organisms. *, **, and *** are shown when the statistical significance (Wilcoxon-Mann-Whitney test, *p*-value) was below 0.05, 0.01, or 0.001, respectively

The emergence of multicellularity and cellular differentiation in cyanobacteria likely required multiple molecular adaptations and innovations, including the regulation of transcription. We investigated whether the distribution of sigma70 genes in the genomes of heterocyst-forming strains (Nostocales) was significantly distinct from that of non-heterocyst organisms (unicellular, filamentous, and unclassified organisms) (Fig. 5A and Additional file 2: Table S3). It was found that families A.1 and F.1 had high numbers of sigma70 homologs on average, regardless of the type of cyanobacteria. Interestingly, the presence of sigma70 proteins in A.1, A.5, C.1, J.1, and K.1 families was notably higher in strains that form heterocysts. The prevalence of members of these five families in such strains might suggest that they are involved in the processes related to the formation or functioning of heterocysts. It is also worth noting that members of the E.1 families are significantly lower in heterocyst-forming organisms. Altogether, these observations reveal that A.1, A.5, C.1, E.1, J.1, and K.1 families are the key sigma70 families that distinguish heterocyst-forming from non-heterocystous organisms.

Analysis was performed on the distribution of the number of homologs per genome for three morphotypes (U, F, and H) for six major sigma70 families (A.1, A.5, C.1, E.1, J.1, and K.1), as shown in Fig. 5B. The results revealed significant biases in the number of sigma70 homologs per genome between U, F, and H for A.1, A.5, E.1, and K.1 families.

Moreover, in the case of C.1 and J.1 families, there were significant differences in the number of sigma70 proteins found in heterocyst organisms compared to unicellular and non-heterocysts forming strains, respectively. These findings (See also Additional file 3: Fig. S5) suggest that sigma70 factors may have contributed to the evolution of cyanobacterial morphotypes and the differentiation process in cyanobacteria. This hypothesis is supported by evidence showing that two sigma factors are involved in heterocyst development in *Nostoc sp.* PCC 7120 [25, 26] and by observations that the formation of akinetes in *Nostoc punctiforme* also involves a sigma factor [27]. Additionally, the formation of hormogonia in *N. punctiforme* is controlled by a regulatory cascade involving three sigma factors [28].

### The evolutionary scenario of sigma70 proteins in cyanobacteria

**Fig. 6:**
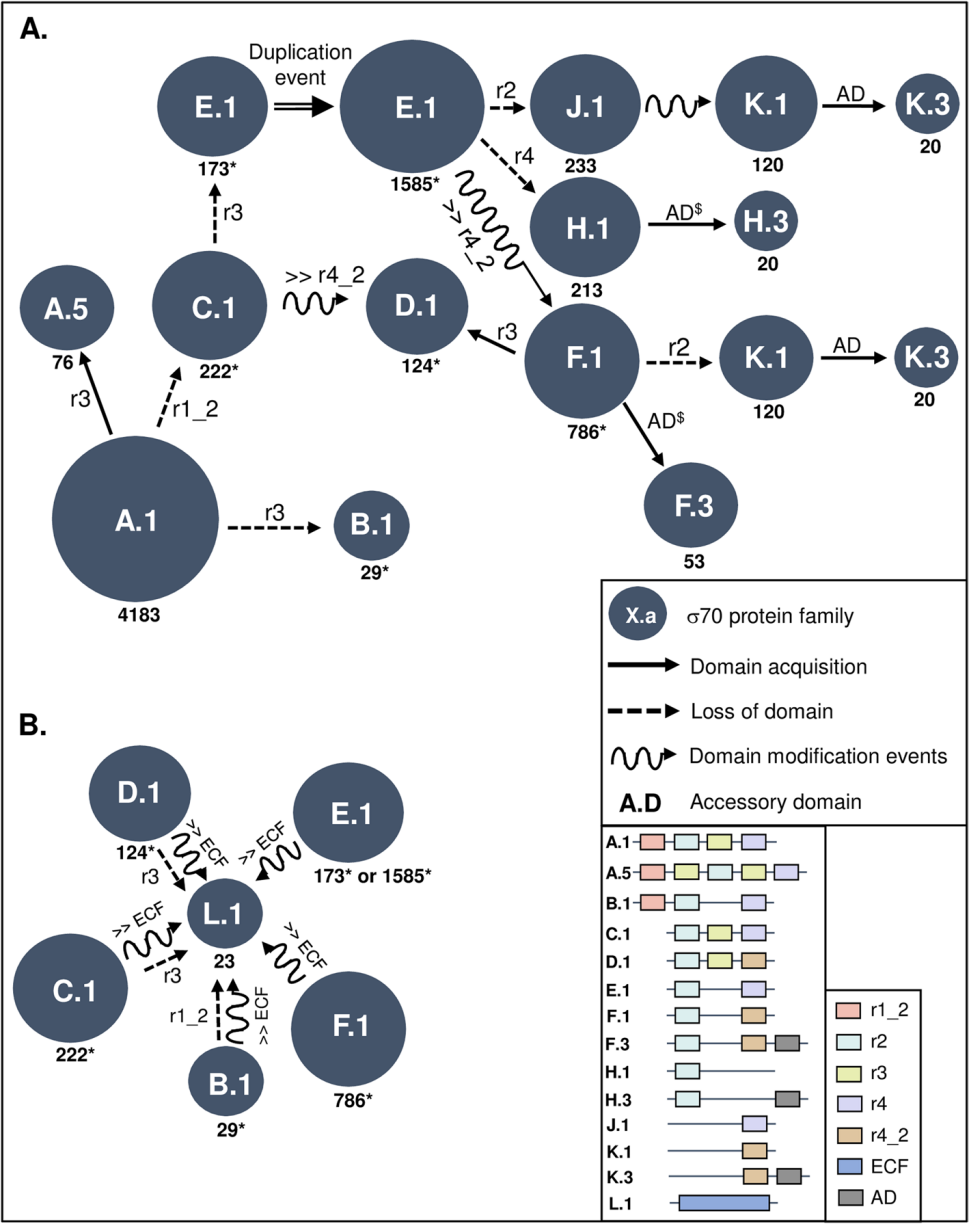
**A** Evolutionary model of sigma families resulting from the A.1 family diversification. **B** Evolutionary model of sigma families resulting from the L.1 family. Protein families are represented by blue circles with the number of homologs at the bottom. Hatched grey circles Plain and dashed arrows correspond to acquisition and loss of domain, respectively. Functional domains r1_2, r2, r3, r4, r4_2, and accessory domains (AD) could be acquired (plain arrows) or lost (dashed arrows) during the evolution. Domain modification events of r4 into r4_2 domain (in panel A) and of r2/r4 or r2/r4_2 into ECF domains (in panel B) are shown. Protein domain organizations are also displayed. $ means that the major AD is DUF6596. * is related to sigma families shared by both scenarios

Two main evolutionary scenarios are proposed for the A.1 family of sigma70 factors in cyanobacteria, based on their wide distribution and domain architecture (Fig. 6A and Additional file 3: Fig. S6). The A.1 family type sigma70 protein (r1.2*r2*r3*r4) was likely present in the cyanobacterial ancestor. Our model suggests that various evolutionary events, such as domain losses, gains, insertions, and modifications, have contributed to the diversification and evolution of sigma70 factors in different cyanobacterial lineages (Fig. 6A). This is the most parsimonious scenario proposed by our model. It is suggested that the A.5 family members would have been generated following a gain of another r3 domain in A.1 family members. On the other hand, the loss of r3 and r1_2 could have generated the B.1 and C.1 families, respectively. The D.1 family could have emerged from C.1 through a substitution within the r4 domain leading to r4_2 in D.1 while E.1 members originated from the loss a r3. Later, the emergence of 1585 sigma70 members from the F.1 (r2*r4_2), F.3 (r2*r4_2*AD), H.1 (r2), H.3 (r2*AD), J.1 (r4), K.1 (r4_2), and K.3 (r4_2*AD) families can be explained. It was first a duplication of E.1 that yielded 1585 members, then followed by various evolutionary events such as r2 and r4 losses, substitutions in r4 (generating r4_2), and/or additional domains (AD) acquisitions. Finally, the D.1 family members could have also emerged via r3 domain insertions in F.1 (Fig. 6A).

The emergence of E.1, F.1, F.3, H.1, H.3, J.1, K.1, and K.3 families can also be explained by a parsimonious scenario that involves a smaller number of members. Additional file 3: Fig. S6 indicates the events of loss, gain, and modification that would have led to the formation of these families. The most plausible hypothesis is that the insertion of the additional domains happened towards the end of the evolutionary process. This is because only a few proteins have these domains, and they are present only at the ends of the proteins.

The L.1 family is suggested to have originated from ancestors belonging to the B.1, C.1, D.1, E.1, or F.1 families (Fig. 6B). ECF (Extracytoplasmic function sigma factor) proteins were previously defined as proteins with the r2 and r4 (or r4_2) domains separated by less than 50 amino acids [29]. This led to the question of whether L.1 members could have been obtained through the loss of r1_2 (from B.1) and r3 domains (from C.1 and D.1), or through the modifications (of both r2 and r4 (or r4_2) domains into ECF) of protein family members such as E.1 and F.1. To answer this question, the sizes of the protein, the total size of protein domain regions, and the protein regions covered by the sigma70 domains were analyzed for each sigma70 family (Additional file 3: Fig. S7, S8 & S9). The results showed that the protein sizes of L.1 members are significantly lower than those of B.1, C.1, D.1, E.1, and F.1, which supports the idea that B.1 members might have an additional step of r2 and r4 modifications to become L.1 members with ECF domain**s **(Additional file 3: Fig. S7). The total sizes of the essential domains along the proteins also showed that C.1 and D.1 are significantly higher than L.1, while E.1 and F.1 are lower compared to L.1 (Additional file 3: Fig. S8). This suggests that L.1 could have resulted from a deletion of the r3 domain within C.1 and D.1 members, followed by the transformation of the remaining domains into ECF domains of L.1. Furthermore, the analysis excluded the simple fusion event of r2 and r4 (or r4_2) domains (in the case of E.1 and F.1), since this event should be followed by amino acid modification of r2 and r4 (or r4_2) domains to form the ECF-specific domain of L.1 members. Indeed, the protein regions covered by essential domains showed that L.1 is significantly higher than E.1 and F.1 but lower than C.1 and D.1 members (Additional file 3: Fig. S9). In conclusion, the specific ECF domain of L.1 family members could have resulted from the r3 deletion and/or r2/r4 modification events in B.1, C.1, D.1, E.1, and F.1 members (as shown in Fig. 6B).

### DUF6596, an accessory domain specific to sigma70 factors

**Fig. 7:**
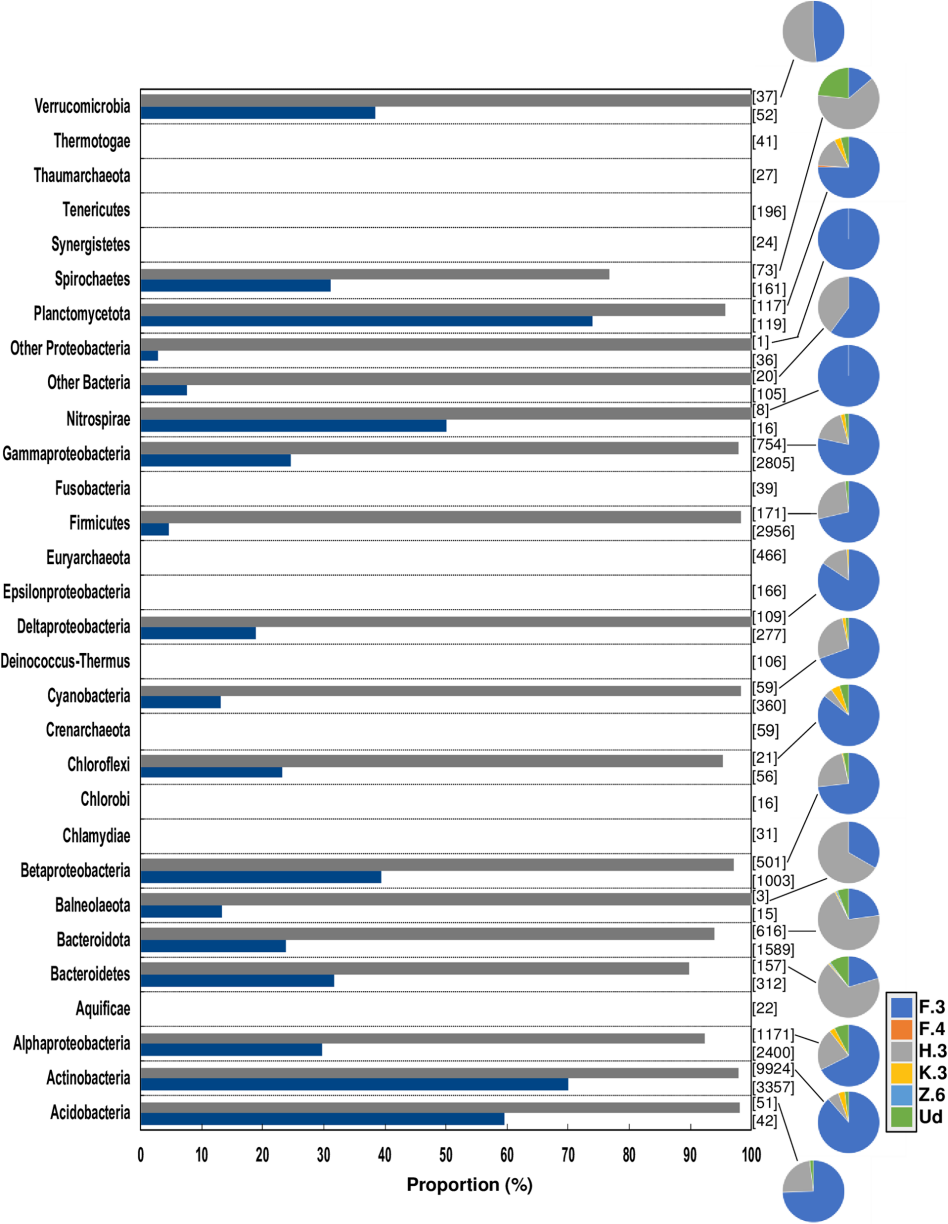
Distribution of DUF6596-containing sigma proteins over the prokaryotic lineages. For each taxonomic clade, dark blue and dark grey bars give the percentage of genomes containing DUF6596 homologs and the percentage of DUF6596 homologs included in sigma70 proteins, respectively. The number of DUF6596-containing sigma proteins (on the top) within the clade as well as the number of analyzed genomes (on the bottom) are shown in brackets. For each clade, pie chart represents the classification and proportion of DUF6596 homologs in sigma families. Pie chart color codes are shown. Ud is related to DUF6596 homologs containing only the DUF6596 protein domains. “Other Bacteria” stands for all other bacteria, excluding Proteobacteria. “Other Proteobacteria” is relative to proteobacterial organisms and combines the smallest clades of proteobacterial classes (See Materials and Methods)

A striking observation among our results is the prevalence of the DUF6596 domain among the sigma70 proteins. It represented 40.5% of the overall accessory domains and was detected in 1.38% of sigma70 homologs in cyanobacteria (Additional file 1: Table S3 and Additional file 2: Table S1). This observation is in line with the association of DUF6596 domains with ECF sigma70 factor proteins in domain databases (https://www.ebi.ac.uk/interpro/entry/InterPro/IPR046531/). This raises the question of a potential conserved evolutionary link between this putative domain and sigma70 factor activity. To test this, we investigated the presence of this domain in proteins of other prokaryotic lineages (See Materials and Methods, Fig. 7, and Additional file 2: Tables S4 & S5). Among the 16,854 prokaryotic genomes (including Cyanobacteria), 30% of them contained DUF6596-encoding genes with more than 60% DUF6596-containing genomes in Acidobacteria, Actinobacteria, and Planctomycetota lineages. Among the DUF6596-containing proteins (13,793 in total), almost 72% of them (representing 9924 proteins) belong to Actinobacteria (Fig. 7). This higher frequency of homologs per genome in Actinobacteria (in comparison to other clades with less than 1.21 homologs per analyzed genome) suggests that this domain might play significant functions in Actinobacteria. This could also mean that Actinobacteria might serve as a key source for the spread of genes encoding this domain through horizontal gene transfer. It is also worth mentioning that no homologs of DUF6596 were detected in Archaea (across 568 genomes) or various bacterial phyla, such as Tenericutes and Epsilonproteobacteria (Fig. 7).

Further analysis revealed that 97% of DUF6596 domains are components of sigma70 proteins, specifically located at their C-terminal extremities (Fig. 7 and Additional file 2: Table S4). As a result, DUF6596 protein homologs were classified into five distinct sigma70 families: F.3, F.4, H.3, K.3, and Z.6, following the sigma70 classification we described above. This pattern mirrors the distribution of sigma70 homologs in cyanobacteria, where sigma70 proteins with DUF6596 accessory domains predominantly belong to the F.3 and H.3 families across other prokaryotic genomes as well. These families account for 76.7–98.0% of all such homologs in prokaryotic clades (Fig. 7 and Additional file 2: Table S5). Collectively, these findings might highlight a significant role of the DUF6596 domain in sigma70 proteins, particularly within actinobacterial lineages where this domain has been uniquely preserved throughout evolution.

## Discussion

Several functional studies have revealed the involvement of sigma70 factors in the adaptive response of cyanobacteria to a variety of stresses and stimuli. Beyond these studies, the classification of these proteins has been based on a limited number of genomes, mainly covering model strains. Therefore, the diversity and distribution of sigma70 factor-encoding genes across the cyanobacterial phylum are largely unknown.

Since sigma70 factors are multidomain proteins, using approaches based solely on direct sequence comparisons — without considering the precise location of domains within proteins (such as BLAST, CLAN-based approaches [30], molecular phylogeny, and protein similarity networks [31] — would introduce biases. These biases could lead to the identification of false positives and the overlooking of true homologs. This is especially relevant given that manual curation methods, like those previously used for sigma70 factor identification, would not have been feasible for this scale of analysis (encompassing 361 genomes and 4193 proteins). Our approach, which combines the search for functional domains with a ranking based on their modularity, is well-suited to the nature of sigma70 factors and the extensive scope of our analysis. For instance, a similar approach has been instrumental in understanding the evolution of domains in protein kinases C [32] and in the neopullulanase subfamily [33]. We used domain modularity to analyze the genomic distribution of sigma70 factors across 361 cyanobacterial genomes, covering the three morphotypes of this phylum (unicellular, filamentous, and filamentous with cell differentiation capability). We also used the domain architectures as a framework to explore the evolution of these factors across the phylum. As a result, we identified 4,193 homologs that we classified into 13 clans and 36 families, 14 of which were considered major given the high prevalence of their members. Compared to previous classification, our strategy of classification can be automatically set up (without manual curation) and therefore adaptable to novel genomes that will appear within the biological databases in the future.

Our results indicate that the primary sigma70 factors belong to the A.1 family (Fig. 3C and Additional file 2: Tables S2 & S3). None of its members possess the r1_1 region, which is conserved in the primary sigma70 factor of *Escherichia coli*. In other words, the homologs classified in the A.1 family in each genome include the primary sigma70 factors of the corresponding strain. It is not possible to distinguish the primary sigma70 factor from the alternative members of this family based on domain structural features alone. Given that the r1_1 domain prevents DNA binding unless the factor is associated with RNA polymerase (RNAP) [34–38], likely, this inhibition is not a critical step in the initiation of transcription within the cyanobacterial phylum. The absence of the r1_1 domain is also consistent with the observed protein lengths, since the canonical *E. coli* protein size is 613 amino acids (aa), while the average size in the novel A family is around 350 aa.

Besides the A.1 family members, the r1_2 domain that facilitates the interaction between RNA polymerase and the template strand [39]was identified exclusively in the A.5 and B.1 family members. This suggests a potentially conserved mechanism across all these sigma70 families, which could permit functional redundancy. The lack of the r2 and r4 domains, which facilitate the interaction between the − 10 and − 35 promoter boxes in certain families (such as K.1, K.3, and L.1), raises doubts about the capability of these proteins to function as RNAP subunits. However, should this indeed be the situation, it would underscore a significant flexibility in the molecular mechanism of cyanobacterial RNAP. It is important to stress that not every cyanobacterial gene promoter possesses both the − 10 and − 35 boxes [21]. This configuration is exclusive to class 1 promoters. In contrast, class 2 promoters contain only the − 10 box, making them recognizable by sigma70 factors that include at least the r2 domain [40, 41], as seen in the H clan sigma70 factors. Class 3 promoters lack both boxes, raising the question of whether they can be transcribed by an RNA polymerase associated with sigma70 factors from the H, J, or K clans (Fig. 3C). Exploring this could shed light on the adaptability of the transcriptional program in these bacteria. Additionally, it is crucial to mention that some fewer common homologs only contain the r1_2 domain (found in the G clan) or the r3 domain (in I clan) (Additional file 1: Table S4). While it seems less probable that these sigma70 factors are functional, investigating their potential roles remains an intriguing prospect.

Interestingly, the variety of several sigma70 factors appears to have been achieved through the addition of different accessory domains, as shown in Fig. 3C and Additional file 1: Tables S3 & S4. To understand the significance of these sigma70 factors, examining how these accessory domains influence their functions would be pertinent. This is especially true for the DUF6596 domain, which is prevalent in genes encoding sigma70 factors not just in cyanobacteria but also across a broader range of prokaryotic genomes (Fig. 7**)**.

A key aspect of our research is derived from examining the distribution of sigma70 factors across different taxonomic groups and morphological types. The data compiled in the heatmap shown in Fig. 4 displays critical insights for subsequent research into how sigma70 factors contribute to cell shape, metabolism, and adaptation to unique environments. For example, the complete lack of sigma70 factors with ECF domains in the Nostocales suggests a direction for future research into their roles. On the other hand, the notable increase in sigma70 factors, particularly within certain families, presents an opportunity to delve into the transcriptional regulation necessary for cell differentiation processes, such as forming heterocysts, hormogonia, and akinetes. Another intriguing observation is the extreme reduction of sigma70 factor diversity in 10 Synechoccocales strains, where only A.1 homologs were found (in 14 Prochlorococcus strains and Synechococcus sp. WH 8119). Investigating what constrains the evolution of these genes in such organisms and how they adjust to their surroundings are important questions to address. For instance, it would be worthwhile to explore if the divergence in their genomic promoters is less pronounced than in other strains.

Multidomain proteins have evolved through deletions, insertions, or modifications of various protein domains. By examining the history of these individual domains, we gain insights into the evolution of these proteins. When applied to sigma70 factor genes found in cyanobacterial genomes, this approach led to the suggestions of an evolutionary narrative for these crucial factors within this group (Fig. 6). The key evolutionary insights related to the sigma70 proteins evolutionary model include: (*i*) a single ancestral origin; (*ii*) the loss of the r1_2 and r3 domains, indicating a relatively lower evolutionary pressure on their functions compared to other domains; (*iii*) the r2 and r4(r4_2) domains are highly conserved evolutionarily, reflecting their essential role in recognizing and binding to target promoters; (*iv*) the insertion of accessory domains appears to be a later evolutionary development; and (*v*) the specific ECF domain likely emerged not merely through a fusion of the r2 and r4(r4_2) domains but through the loss of the r1_2 and r3 domains followed by a specialized adaptation of the r2/r4(r4_2) domains. The upcoming challenge involves understanding the effect of modularity on the functionality of sigma70 factors and examining how their diversification has shaped the evolutionary trajectory of these organisms.

## Materials and methods

### Datasets

The genome data of 361 cyanobacterial strains available in January 2022 were downloaded from NCBI ftp site (ftp:/ftp.ncbi.nih.gov/genomes/) and were used as the primary data source (Additional file 2: Table S2). This data source includes all complete genomes in the highest levels of assembly [mostly as “Complete” or “Chromosome” (~ 65% of the total) status as defined at the NCBI], and if not [as “Scaffold” or “Contig” status but limited to “Reference” or “Representative” genomes] (Additional file 3: Fig. S10). These data also included all gene annotation features as well as organism taxonomy lineages. Morphological and physiological traits (or phenotypical traits) of cyanobacteria organisms were provided by the literature [11, 42–44] and their distributions are shown in Additional file 3: Fig. S11. The studied organisms belong to the overall diversity of cyanobacteria (11 main taxonomic orders) for which 45.1%, 21.8%, and 32.4% are unicellular (U), filamentous without heterocysts (F) and filamentous and heterocyst-forming (H) organisms, respectively. Note that the two unclassified cyanobacteria organisms (cyanobacterium endosymbiont of Epithemia turgida isolate EtSB Lake Yunoko and cyanobacterium endosymbiont of Rhopalodia gibberula) in our data were arbitrarily set to unicellular organisms.

Hidden Markov Models (HMMs) profiles of protein domain families (Pfam 34.0, February 2022) was downloaded from the ftp.ebi.ac.uk/pub/databases/Pfam/releases/ ftp site. Sigma70 proteins (from Cyanobacteria and *E. coli* organisms) used as representative seed proteins were downloaded from the Uniprot database (www.uniprot.org) (Additional file 1: Table S1). Comparative analysis of proteins containing the DUF6596 domain among prokaryotes required the additional download of 16,494 reference/representative and complete prokaryotic genomes (Bacteria and Archaea, but without Cyanobacteria; from NCBI ftp site, December 2022) (Additional file 2: Table S6). To ensure consistency in our distribution analysis, we only included prokaryotic phyla that had 15 or more organisms. Phyla with fewer organisms were combined into a single group called “Other Bacteria” without proteobacterial organisms. Proteobacteria classes such as Gammaproteobacteria, which have a large number of genomes, were analyzed as phylum clades. Otherwise, they were combined into a group called “Other Proteobacteria”.

### Identification, classification, and distribution of homologs

HMMER3 package [45] and HMM profiles were first used to establish the list of seed functional domains associated with representative seed proteins from representative cyanobacteria and *E. coli* (Additional file 1: Table S2). Alignments with a score higher than the Pfam trusted thresholds were considered significant seed domains. This led to 7 (Sigma70_r1_1, Sigma70_r1_2, Sigma70_r2, Sigma70_r3, Sigma70_r4, Sigma70_r4_2, and Sigma70_ECF) representative seed domains from Pfam domain databases. HMMER3 and self-written Perl scripts were then used to search for sigma70 protein homologs (with representative seed domains) in cyanobacterial genomes. For each representative protein, the presence of at least one seed domain was chosen as a requisite to identify putative sigma70 homologs. As described above, alignments with a score higher than the Pfam thrust thresholds were also considered significant and in case of overlapping domains, the longest ones with the best E-values were chosen. All putative sigma70 homologs retrieved were subsequently analyzed with HMMER3 package to determine the potential presence of additional functional domains. In-house Perl scripts were used to define the domain organization of each homolog. A final set of sigma70 homologs was selected based on the strict presence of at least one seed domain. This led to the identification of sigma70 homolog domain architectures. Each homolog was then classified into a clan and family based on the domain architecture. To name each sigma70 factor unit, a novel nomenclature was proposed. The distribution of sigma70 homologs among cyanobacterial genomes was mainly examined based on their presence or absence in each genome, as well as taxonomic order and morphotype (U, F, H). The same procedure was used to identify DUF6596-containing proteins in the genomes of prokaryotes (excluding Cyanobacteria), using the HMMER3 package and DUF6596 profile for the requisite seed domains.

### Statistical analysis

Wilcoxon-Mann-Whitney, one-sample *t-*tests, as well as boxplot, heatmap, and distribution graphs, were done using *R* [46]. For statistical analysis, the Shapiro-Wilk test (with a 5% error) was used to assess the normal distribution of our data. In the case of normal distribution, the Student *t-*test (with a 5% error) was applied. Otherwise, the non-parametric Wilcoxon-Mann-Whitney tests were used (with 5% error). For boxplot analysis, taxonomic orders with a low number of genomes (< 10) (i.e., Chroococcidiopsidales, Gloeobacterales, Gloeoemargaritales, Pleurocapsales, Spirulinales, Thermostichales and unclassified Cyanobacteria) were fused into a single group (named Others) before computation.

### Supplementary Information

Additional file 1.

Additional file 2.

Additional file 3.

## Data Availability

All data generated or analyzed during this study are included in this published article. The accession numbers of genome datasets used during the current study are provided in Additional file 2: Tables S2 & S6.

## Abbreviations

AD: Accessory domain
BLAST: Basic local alignment sequence tool
DUF: Domain of Unknown Function
ECF: Extracytoplasmic factor
HMM: Hidden Markov Model
RNAP: RNA polymerase
