# Supplementary material for: The modular architecture of sigma factors in cyanobacteria: a framework to assess their diversity and understand their evolution
Source: BMC Genomics. 2024 May 24;25:512. doi: 10.1186/s12864-024-10415-x (PMC11119718; doi:10.1186/s12864-024-10415-x)
Supplement: Supplementary file 1 — Additional file 1. [file 12864_2024_10415_MOESM1_ESM.pdf]

Additional file 1:

Table S1. Sigma70 protein seeds (or representative sigma proteins) used in this study. To ensure the taxonomy diversity of the protein seeds, the latter belongs to 8 cyanobacterial species including *Anabaena* sp. PCC 7120, *Anabaena variabilis* PCC 7937, *Synechococcus* sp. PCC 7002, *Synechococcus elongatus* PCC 7942, *Synechocystis* PCC 6803, *Microcystis aeruginosa* NIES-843, *Nostoc punctiforme* PCC 73102, and *Thermosynechococcus vestitus* BP-1 as well as *Escherichia coli* K12 with the canonical RpoD protein. Protein seed accession numbers are from Uniprot database with the gene names in parenthesis when available. Current sigma70 families (from SigA to SigR) as described in the literature[1, 2]are shown. Gene names are in bold if distinct from the literature family name. For each seed protein. the domain patterns were determined (using Pfam database, http://pfam.xfam.org/) as described in **Materials and Methods**. Domain abbreviations are: r1_1, for Sigma70_r1_1; r1_2, Sigma70_r1_2; ner, Sigma70_ner; r2, Sigma70_r2; r3, Sigma70_r3; r4, Sigma70_r4; and r4_2, Sigma70_r4_2. Functional descriptions of the domains are shown in **Additional file 1: Table S2**. The alternance of light grey and white colors facilitates reading. Note that *Thermosynechococcus elongatus* BP-1 and *Anabaena variabilis* PCC 7937 were renamed as *Thermosynechococcus vestitus* BP-1 and *Trichormus variabilis* PCC 7937, respectively. When available the primary gene name (from Uniprot database) is shown in parenthesis at the bottom of the accession number.

Organisms	Uniprot accession numbers	Sizes (AA)	Domain patterns (Pfam)	Curent family names
Escherichia coli K12	P23484 (FecI)	173	r2*r4_2	**σ19**
	P0AGB6 (RpoE)	191	r2*r4_2	**σ24**
	P0AEM6 (RpoF)	239	r2*r3*r4	**σ28**
	P0AGB3 (RpoH)	284	r2*r4	**σ32**
	P13445 (RpoS)	330	r1_2*r2*r3*r4	**σ38**
	P00579 (RpoD)	613	r1_1*r1_2*ner*r2*r3*r4	**σ70**
Synechocystis sp. PCC 6803	P74565 (SigA)	425	r1_2*r2*r3*r4	**SigA**
Microcystis aeruginosa NIES-843	B0JGK3 (SigA)	416	r1_2*r2*r3*r4	
Synechococcus sp. PCC 7002	Q59965 (SigA)	375	r1_2*r2*r3*r4	
Synechococcus elongatus PCC 7942	P38023 (SigA1)	399	r1_2*r2*r3*r4	
Thermosynechococcus vestitus BP-1	Q8DL79 (SigA)	418	r1_2*r2*r3*r4	
Anabaena sp. PCC 7120	P26683 (SigA)	390	r1_2*r2*r3*r4	

Synechocystis sp. PCC 6803	Q55525 **(RpoD)**	345	r1_2*r2*r3*r4	**SigB**
Microcystis aeruginosa NIES-843	B0JU57	328	r1_2*r2*r3*r4	
Synechococcus sp. PCC 7002	B1XKF5 (SigB)	328	r1_2*r2*r3*r4	
Synechococcus elongatus PCC 7942	Q31ME3 **(SigA2)**	320	r1_2*r2*r3*r4	
Thermosynechococcus vestitus BP-1	Q8DKM8 (SigB)	309	r1_2*r2*r3*r4	
Anabaena sp. PCC 7120	Q03065 (SigB)	332	r1_2*r2*r3*r4	
Anabaena sp. PCC 7120	Q9KHE5 **(SigE)**	327	r1_2*r2*r3*r4	
Anabaena sp. PCC 7120	Q8ZSA3	323	r1_2*r2*r3*r4	
Anabaena sp. PCC 7120	Q8YKW1	316	r1_2*r3*r2*r3 *r4	
Synechocystis sp. PCC 6803	Q59996 (SigC)	404	r1_2*r2*r3*r4	**SigC**
Microcystis aeruginosa NIES-843	B0JRK7	414	r1_2*r2*r3*r4	
Synechococcus PCC 7002	B1XMU3 (SigC)	398	r1_2*r2*r3*r4	
Synechococcus elongatus PCC 7942	Q31M40	398	r1_2*r2*r3*r4	
Thermosynechococcus vestitus BP-1	Q8DLJ2 (SigC)	375	r1_2*r2*r3*r4	
Anabaena sp. PCC 7120	Q03066 (SigC)	416	r1_2*r2*r3*r4	
Nostoc punctiforme PCC 73102	B2IV51	416	r1_2*r2*r3*r4	
Synechocystis sp. PCC 6803	P73666 **(RpoD)**	318	r1_2*r2*r3*r4	**SigD**
Microcystis aeruginosa NIES-843	B0JTP4 (SigD)	318	r1_2*r2*r3*r4	
Synechococcus sp. PCC 7002	B1XQ38 **(RpoD)**	318	r1_2*r2*r3*r4	
Synechococcus elongatus PCC 7942	Q31QG5 (**SigA3**)	320	r1_2*r2*r3*r4	
Thermosynechococcus vestitus BP-1	Q8DM59 (SigD)	315	r1_2*r2*r3*r4	
Anabaena sp. PCC 7120	Q9KHE8 (SigD)	332	r1_2*r2*r3*r4	
Synechocystis sp. PCC 6803	P73710 **(RpoD)**	369	r1_2*r2*r3*r4	**SigE**
Microcystis aeruginosa NIES-843	B0JY74 (SigE)	377	r1_2*r2*r3*r4	
Synechococcus sp. PCC 7002	B1XNJ6 (SigE)	365	r1_2*r2*r3*r4	

Anabaena sp. PCC 7120	Q9KHE4 **(SigF)**	390	r1_2*r2*r3*r4	
Synechocystis sp. PCC 6803	P74595 **(RpoF)**	258	r2*r4	SigF
Microcystis aeruginosa NIES-843	B0JY76 (SigF)	258	r2*r3*r4_2	
Synechococcus sp. PCC 7002	Q9WWP8 (SigF)	259	r2*r3*r4	
Synechococcus elongatus PCC 7942	Q31N29 (SigF)	259	r2*r3*r4	
Thermosynechococcus vestitus BP-1	Q8DKW3 (SigF)	261	r2*r3*r4_2	
Anabaena sp. PCC 7120	Q8YQH5	258	r2*r3*r4	
Nostoc punctiforme PCC 73102	B2IZ53	269	r2*r3*r4	
Synechocystis sp. PCC 6803	P73431 **(SigE)**	223	r2*r4_2	SigG
Microcystis aeruginosa NIES-843	B0JMU0 **(SigE)**	218	r2*r4_2	
Synechococcus sp. PCC 7002	Q9WWP6 (SigG)	218	r2*r4_2	
Synechococcus elongatus PCC 7942	Q31LW6	218	r2*r4_2	
Thermosynechococcus vestitus BP-1	Q8DH51 (SigG)	219	r2*r4_2	
Anabaena sp. PCC 7120	Q8YS12 **(SigE)**	218	r2*r4_2	
Nostoc punctiforme PCC 73102	B2J7T6	218	r2*r4_2	
Synechocystis sp. PCC 6803	P73744 **(SigE)**	186	r2*r4	SigH
Synechococcus sp. PCC 7002	Q9WWP4 (SigH)	199	r2*r4_2	
Thermosynechococcus vestitus BP-1	Q8DM09 (SigH)	201	r2*r4_2	
Synechocystis sp. PCC 6803	Q55192	185	r2*r4	SigI
Synechococcus elongatus PCC 7942	Q31LN5	196	r2*r4	
Anabaena sp. PCC 7120	Q8YUY8	201	r2*r4_2	
Synechococcus elongatus PCC 7942	Q31MA5 **(SigF)**	260	r2*r4_2	SigJ
Anabaena sp. PCC 7120	Q8Z026	261	r2*r4_2	
Nostoc punctiforme PCC 73102	B2IYG2	261	r2*r4_2	
Nostoc punctiforme PCC 73102	B2J640	434	r2*r4_2*DUF6596	**SigK**
Nostoc punctiforme PCC 73102	B2J000	772	r4_2*NB-ARC*TPR_MalT	**SigL**
Synechococcus elongatus PCC 7942	Q31QR8 **(SigA4)**	311	r1_2*r2*r3*r4	

Synechococcus elongatus PCC 7942	Q31MY2	310	r1_2*r2*r3*r4	**SigM**
Trichormus variabilis PCC 7937	Q3M2U9	240	r2	**SigN**
Trichormus variabilis PCC 7937	Q3M2G3	314	r1_2*r2*r3*r4	**SigP**
Nostoc punctiforme PCC 73102	B2JBU3	396	r4_2	**SigR**

Table S2. Description of HMM profile domains associated with sigma70 protein seeds. HMM profiles and their functional descriptions are from the Pfam database (http://pfam.xfam.org/), and the literature [3]. Profile domains were classified into two categories: essential domains, known to have crucial roles in the transcription initiation, and accessory domains. Pfam accession numbers of the domain profiles are in parenthesis. Domain abbreviations are: r1_1, Sigma70_r1_1; r1_2, Sigma70_r1_2; ner, Sigma70_ner; r2, Sigma70_r2; r3, Sigma70_r3; r4, Sigma70_r4; r4_2, Sigma70_r4_2; and ECF, Sigma70_ECF.

	**Short domain names**	**## of positions**	**Functional descriptions**
	**r1_2** (PF00140)	34	σ1.2. region 1.2; promotes the the interaction of the RNA polymerase complex and non-template strand.
	**r2** (PF04542)	205	σ2. region 2; promotes attachment to the -10 promoter region
	**r3** (PF04539)	71	σ3. region 3; promotes the stabilization of the DNA and the RNA polymerase complex.
	**r4** (PF04545)	78	σ4. region 4; promotes attachment to the -35 promoter region
	**r4_2** (PF08281)	50	σ4.2. region 4; promotes attachment to the -35 promoter region
	**ECF** (PF07638)	185	Probably RNA polymerase sigma factors belonging to the extra-cytoplasmic function (ECF) subfamily and display sequence similarity to r1_2 and r4.
**Accessory domains**	**r1_1** (PF03979)	82	σ1.1. region 1.1; prevent non-specific interactions with DNA promoters
	**ner** (PF04546)	205	σ_ner. non-essential region – the function of this domain is unclear and can be removed without loss of function
	**DUF6596** (DUF6596)	102	Domain of unknown function (DUF)
	**NB-ARC** (PF00931)	252	NB-ARC domain
	**TPR_MalT** (PF17874)	336	MalT-like TPR region

Table S3. Functional descriptions of accessory protein domains located within sigma70 homologs. Short names and functional descriptions of accessory domains were taken from the Pfam database (http://pfam.xfam.org/). The proportion of protein domains (in %) was calculated from the total number of domains. Higher proportions (>2% of the total) of domains are shaded in light grey. * stands for accessory domains located within the seed proteins. Detailed roles of some of these domains are: WD40 are short Trp-Asp (W-D) dipeptide repeats which are widely found in proteins that are involved in a wide variety of cellular processes such as signal transduction and transcription [4, 5]; zf-C4_ClpX which is a molecular domain, the function of which is not well known, located within the ClpX protein [6, 7], an ATPase acting as the substrate of the ClpXP protease and as a chaperone; and Ank_2 is a functional domain with Ankyrin-like repeats (tandemly repeated modules of about 33 amino acids). Ank repeats have been found in proteins of diverse functions such as transcriptional initiators, cell-cycle regulators cytoskeletal, ion transporters, and signal transducers; and might be the results of horizontal gene transfer in prokaryotes [8]. the AAA_35 domain is a functional domain located in AAA-type ATPases [9]; and finally, the CRISPR_Cas6/CRIPSPR_Cas6_N (N-ter) domains that structure the Cas6 protein which is involved in the synthesis of guide RNAs [10].

Short domain names	Functional descriptions	Proportions (%)
AAA_16	AAA ATPase domain	0.70
AAA_35	AAA-like domain	2.80
ANAPC4_WD40	Anaphase-promoting complex subunit 4. WD40 domain	0.70
Ank_2	Ankyrin repeats (3 copies)	5.59
Ank_4	Ankyrin repeats (many copies)	1.40
CBS	CBS domain	1.40
CRISPR_Cas6	CRISPR-associated endoribonuclease Cas6	2.10
CRISPR_Cas6_N	CRISPR Cas6 N-terminal domain	2.10
DUF6596*	Family of unknown function (DUF6596)	40.56
FGE-sulfatase	Sulfatase-modifying factor enzyme 1	0.70
Flavodoxin_2	Flavodoxin-like fold	0.70
HTH_23	Homeodomain-like domain	1.40
NACHT	NACHT domain	0.70
NB-ARC*	NB-ARC domain	9.09
NERD	Nuclease-related domain	0.70
Response_reg	Response regulator receiver domain	0.70
RskA	Anti-sigma-K factor rskA	0.70
SnoaL_2	SnoaL-like domain	0.70
SnoaL_3	SnoaL-like domain	0.70
SpvB	Salmonella virulence plasmid 65kDa B protein	0.70
TauE	Sulfite exporter TauE/SafE	0.70
TPR_1	Tetratricopeptide repeat	0.70
TPR_4	Tetratricopeptide repeat	1.40
TPR_8	Tetratricopeptide repeat	0.70
TPR_11	TPR repeat	2.10
TPR_12	Tetratricopeptide repeat	1.40
TPR_MalT*	MalT-like TPR region	4.20
WD40	WD domain. G-beta repeat	8.39
zf-C4_ClpX	ClpX C4-type zinc finger	6.29

Table S4. Classification of cyanobacterial sigma70 proteins into clans and families. Sigma70 homologs were classified into clans [A to L] and Z (unclassified clan of homologs), and each clan was subclassified into families with identical patterns (core patterns) (in bold). In each pattern. functional domains are separated with an "*". Major sigma70 cyanobacteria families (i.e., with at least 20 homologs) are shaded in light grey. The number of homologs as well as their proportions relative to the number of homologs in the clan and in the aggregate are shown. Abbreviations of essential domains are: r1_2, Sigma70_r1_2; r2, Sigma70_r2; r3, Sigma70_r3; r4, Sigma70_r4; and r4_2, Sigma70_r4_2. The description domains are shown in **Additional file 1: Tables S2** & **S3**. For the main families, the number of genomes is shown in brackets. $ stands for the total number of genomes deserved by the entire family. Note that r1_1 and ner domains were considered in this classification as accessory domains.

Clans	Families	Domain patterns	## of homologs	% (clan)	% (total)
A	A.1	**r1_2*r2*r3*r4**	2030 [361]	96.20	48.41
	A.2	r1_1***r1_2*r2*r3*r4**	1	0.05	0.02
	A.4	NERD***r1_2*r2*r3*r4***TPR_11*TPR_11*TPR_11*TPR_1*TPR_1	1	0.05	0.02
	A.5	**r1_2*r3*r2*r3*r4**	76 [65]	3.60	1.81
	A.6	**r1_2***ner***r2*r3*r4**	1	0.05	0.02
	A.7	**r1_2*r2*r3*r3*r4**	1	0.05	0.02
B	B.1	**r1_2*r2*r4**	29 [27]	100	0.69
C	C.1	**r2*r3*r4**	223 [210]	99.55	5.32
	C.5	**r2***r3***r3*r4**	1	0.45	0.02
D	D.1	**r2*r3*r4_2**	124 [109]	100	2.96
E	E.1	**r2*r4**	174 [118]	99.43	4.15
	E.3	**r2*r4***zf-C4_ClpX	1	0.57	0.02
F	F.1	**r2*r4_2**	788 [308]	93.47	18.80
	F.2	SpvB***r2*r4_2**	1	0.12	0.02
		Flavodoxin_2*SnoaL_3***r2*r4_2**	1	0.12	0.02
	F.3	**r2*r4_2***Ank_2	1	0.12	0.02
		**r2*r4_2***Ank_2*Ank_2	1	0.12	0.02
		**r2*r4_2***Ank_2*Ank_2*Ank_2*Ank_4	1	0.12	0.02
		**r2*r4_2***Ank_2*Ank_2*Ank_4	1	0.12	0.02
		**r2*r4_2***CBS*CBS	1	0.12	0.02
		**r2*r4_2***DUF6596	41 [43$^{\$}$]	4.86	0.98
		**r2*r4_2***SnoaL_2	1	0.12	0.02
		**r2*r4_2***zf-C4_ClpX	6	0.71	0.14
G	G.1	**r1_2**	17	100	0.40
H	H.1	**r2**	213 [137]	91.42	5.08
	H.3	**r2***DUF6596	15 [20$^{\$}$]	6.44	0.36
		**r2***DUF6596*TPR_8	1	0.43	0.02
		**r2***HTH_23	2	0.86	0.05
		**r2***RskA	1	0.43	0.02
		**r2***TauE	1	0.43	0.02
I	I.1	**r3**	5	100	0.12
J	J.1	**r4**	233 [131]	97.90	5.56
	J.2	FGE-sulfatase***r4**	1	0.42	0.02
		TPR_12*TPR_12***r4**	1	0.42	0.02
	J.3	**r4***AAA_35	1	0.42	0.02
		**r4***r4_2	1	0.42	0.02
		**r4***zf-C4_ClpX	1	0.42	0.02
K	K.1	**r4_2**	120 [92]	83.33	2.87
	K.2	CRISPR_Cas6_N*CRISPR_Cas6***r4_2**	3	2.08	0.07
	K.3	**r4_2***AAA_16	1	0.69	0.02
		**r4_2***AAA_35	3	2.08	0.07
		**r4_2***DUF6596	1	0.69	0.02
		**r4_2***NACHT*ANAPC4_WD40*WD40*WD40*WD40*WD40*WD 40*WD40*WD40*WD40*WD40*WD40*WD40	1	0.69	0.02
		**r4_2***NB-ARC	6 [19$^{\$}$]	4.17	0.14
		**r4_2***NB-ARC*TPR_4*TPR_4	1	0.69	0.02
		**r4_2***NB-ARC*TPR_MalT	6	4.17	0.14
		**r4_2***r4_2	1	0.69	0.02
		**r4_2***zf-C4_ClpX	1	0.69	0.02
L	L.1	**ECF**	23 [19]	95.83	0.55
	L.2	Response_reg***ECF**	1	4.17	0.02
Z	Z.1	r1_2*r2	6	22.22	0.14
	Z.2	r1_2*r2*r3	3	11.11	0.07
	Z.3	r1_2*r3	1	3.70	0.02
	Z.4	r1_2*r3*r2	1	3.70	0.02
	Z.5	r1_2*r3*r2*r3	1	3.70	0.02

	Z.6	r2*ECF	2	7.41	0.05
	Z.7	r2*r3	4	14.81	0.10
	Z.8	r3*r2*r3	1	3.70	0.02
	Z.9	r3*r4	8	29.93	0.19
			**4193**	**-**	**100**

References

1. Imamura S, Asayama M. Sigma Factors for Cyanobacterial Transcription. Gene Regul Syst Bio. 2009;3:GRSB.S2090.
2. Srivastava A, Summers ML, Sobotka R. Cyanobacterial sigma factors: Current and future applications for biotechnological advances. Biotechnol Adv. 2020;40:107517.
3. Paget MS. Bacterial sigma factors and anti-sigma factors: Structure, function and distribution. Biomolecules. 2015;5:1245–65.
4. Xu C, Min J. REVIEW Structure and function of WD40 domain proteins. Protein Cell. 2011;2:202–14.
5. Jain BP, Pandey S. WD40 Repeat Proteins: Signalling Scaffold with Diverse Functions. Protein J. 2018;37:391–406.
6. Banecki B, Wawrzynow A, Puzewicz J, Georgopoulos C, Zylicz M. Structure-Function Analysis of the Zinc-binding Region of the ClpX Molecular Chaperone. Journal of Biological Chemistry. 2001;276.
7. Gamsjaeger R, Liew CK, Loughlin FE, Crossley M, Mackay JP. Sticky fingers: zinc-fingers as protein-recognition motifs. Trends in Biochemical Sciences. 2007;32.
8. Bork P. Hundreds of ankyrin-like repeats in functionally diverse proteins: Mobile modules that cross phyla horizontally? Proteins: Structure, Function, and Bioinformatics. 1993;17:363–74.
9. Puchades C, Sandate CR, Lander GC. The molecular principles governing the activity and functional diversity of AAA+ proteins HHS Public Access. Nat Rev Mol Cell Biol. 2020;21:43–58.
10. Makarova KS, Wolf YI, Iranzo J, Shmakov SA, Alkhnbashi OS, J Brouns SJ, et al. Evolutionary classification of CRISPR-Cas systems: a burst of class 2 and derived variants substantially contributed to discussion of the content. Nat Rev Microbiol. 2020;15:67–83.
