# Supplementary material for: The modular architecture of sigma factors in cyanobacteria: a framework to assess their diversity and understand their evolution
Source: BMC Genomics. 2024 May 24;25:512. doi: 10.1186/s12864-024-10415-x (PMC11119718; doi:10.1186/s12864-024-10415-x)
Supplement: Supplementary file 3 — Additional file 3. [file 12864_2024_10415_MOESM3_ESM.pdf]

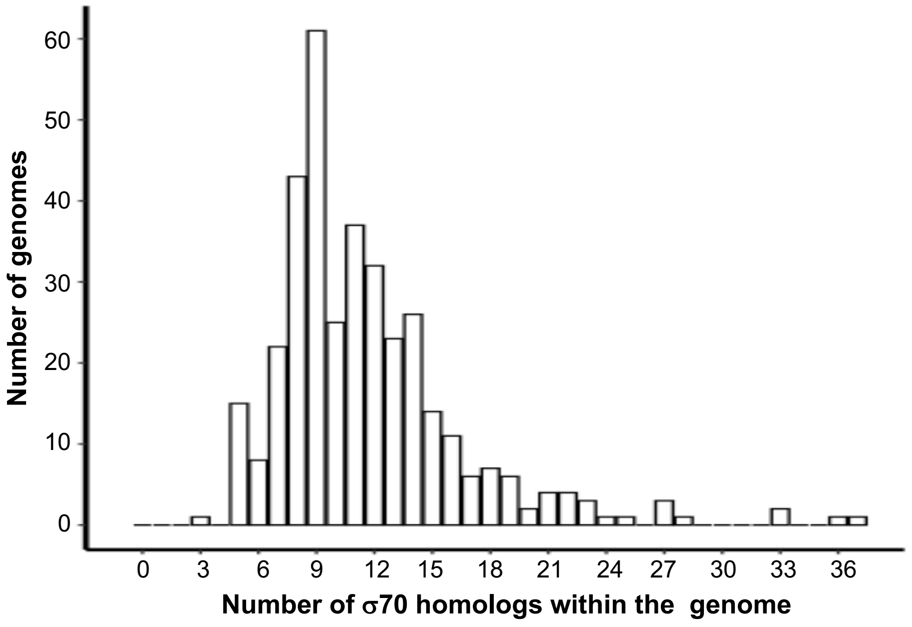

Fig. S1
Proportion of the cyanobacterial genomes as a function of the number of sigma70 homologs per genome.

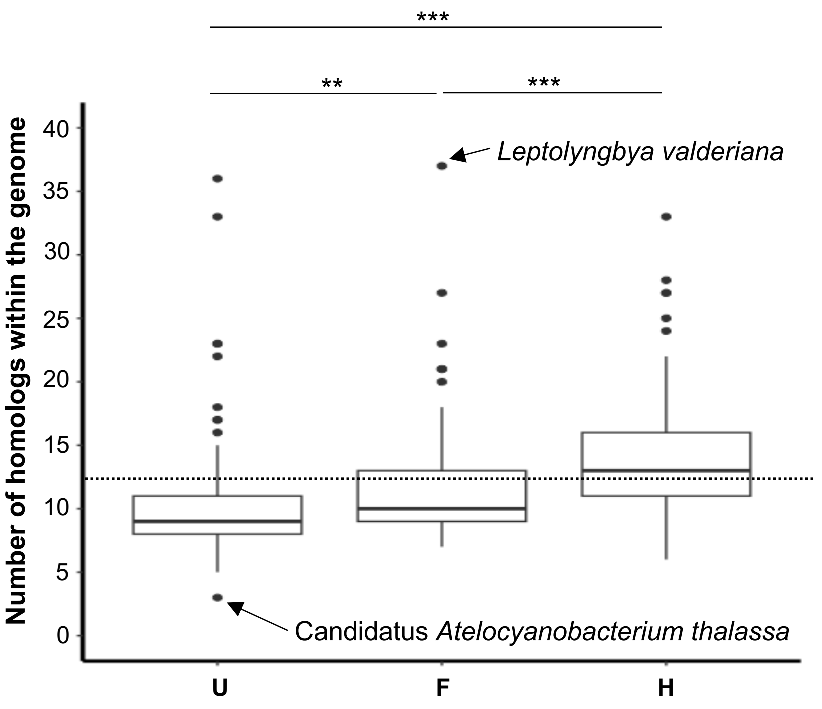

Fig. S2

Distribution of the number of sigma70 homologs within genomes as a function of the phenotypic/physiological traits.

U, unicellular; F, filamentous non-heterocyst-forming; and H, heterocyst-forming organisms. The dashed line represents the average number of homologs in genome among all organisms. ** and *** are shown when the statistical significance (Wilcoxon-Mann-Whitney test, *p*-value) was below 0.01 or 0.001, respectively.

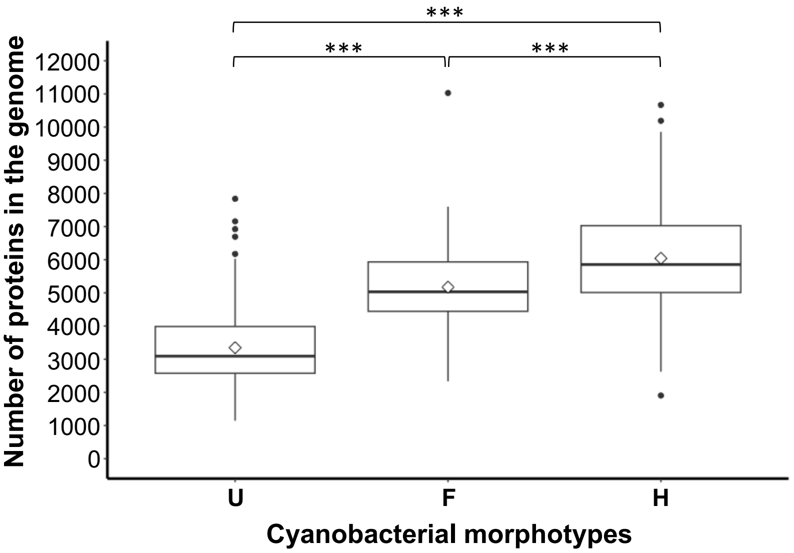

Fig. S3

Distribution of the number of proteins in the genomes of cyanobacterial morphotypes. U, unicellular; F, filamentous non-heterocyst-forming; and H, heterocyst-forming organisms. Square diamonds are relative to the means of the distribution. *** is shown when the statistical significance (Wilcoxon-Mann-Whitney test, *p*-value) was below 0.001.

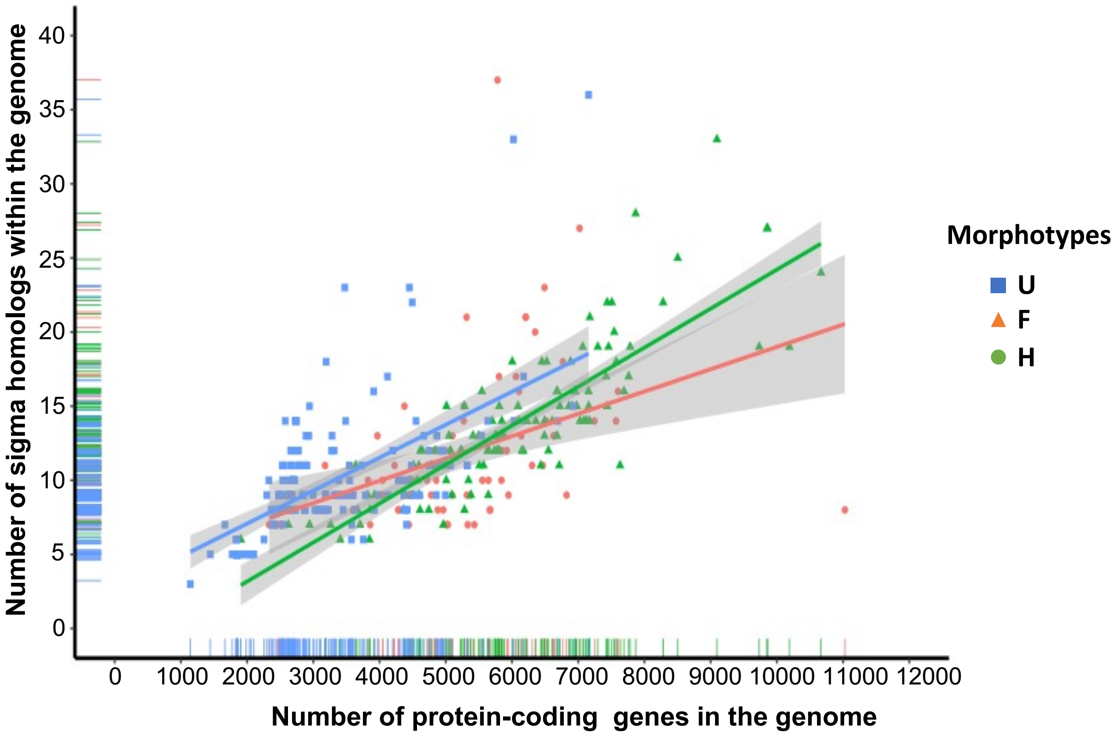

Fig. S4

Number of sigma homologs in function of the number of proteins in the genomes of cyanobacterial morphotypes.

U, unicellular; F, filamentous non-heterocyst-forming; and H, heterocyst-forming organisms. Blue square, pink dot, and green triangle point data are relative to U, F and H morphotypes, respectively. Blue, pink and green lines represent the linear regression lines (with 95% confidence regions in grey) associated with U, F, and H data, respectively. The marginal rug plot (with the same color codes) was added to the scatter plot in order to visualize the distribution of data on each axis.

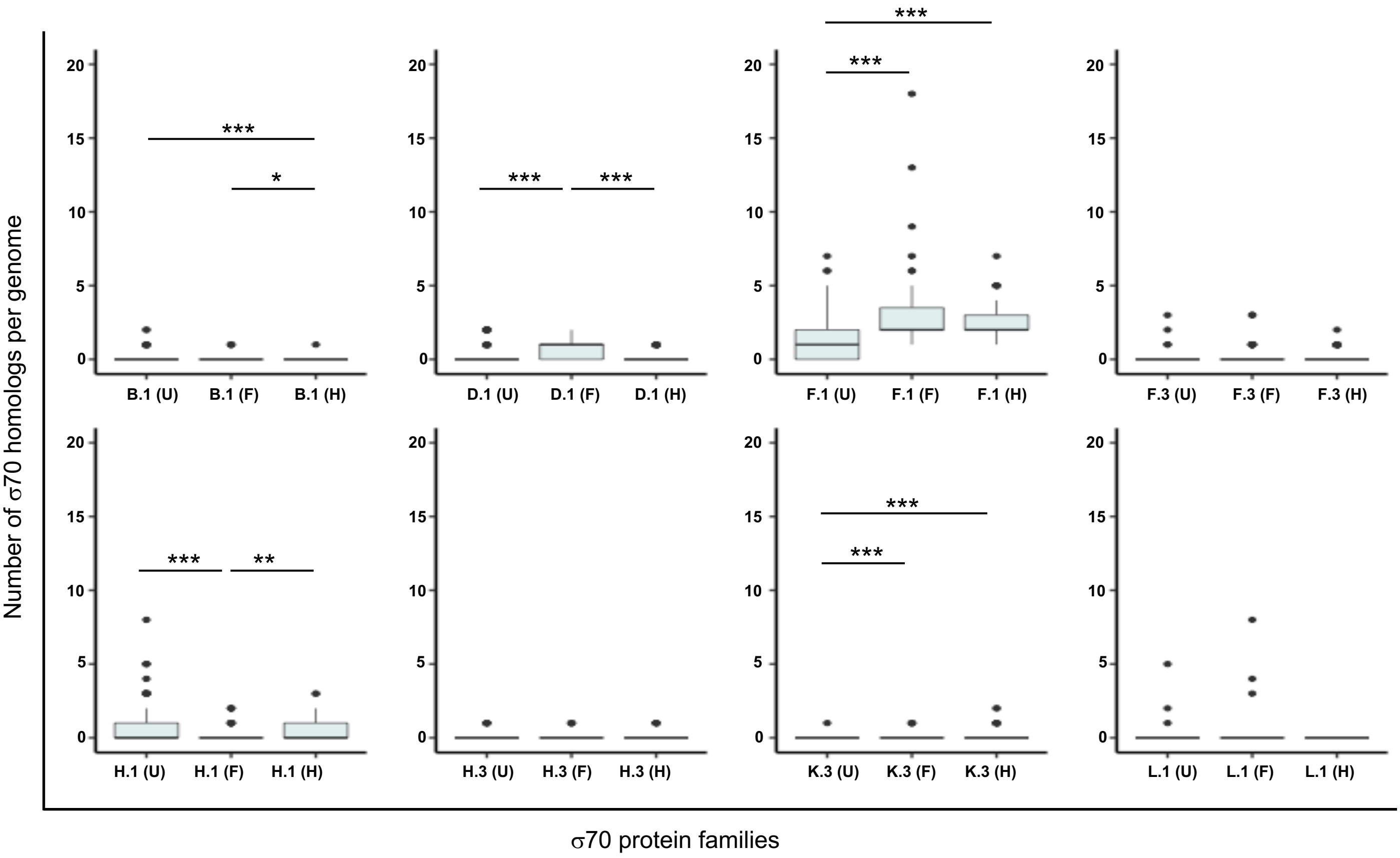

Fig. S5

Distribution of the number of homologs per genome as a function of the protein families and the morphological, and physiological traits. Morphological and physiological traits are: U, unicellular; F, filamentous non-heterocyst-forming; and H, heterocyst-forming organisms. For each protein family, all possible comparisons were performed between U, F, and H organisms. *, **, and *** are shown when the statistical significance (Wilcoxon-Mann-Whitney test, *p*-value) was below 0.05, 0.01, or 0.001, respectively.

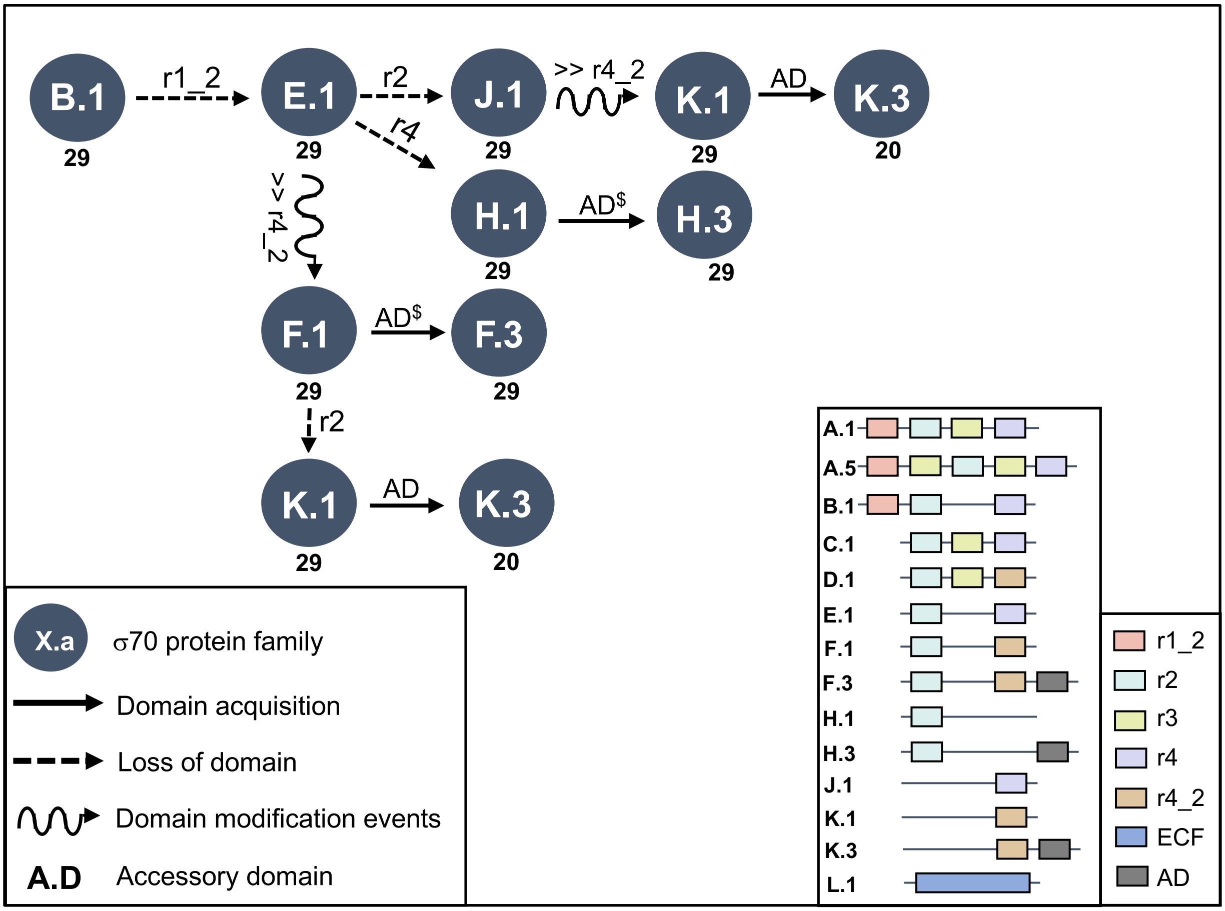

Fig. S6

Evolutionary model of sigma proteins in cyanobacteria with the less parsimonious scenario that generate the main sigma families (except L.1). Protein families are represented by blue circles with the number of homologs at the bottom. Plain and dashed arrows correspond to acquisition and loss of domain, respectively. Functional domains r1_2, r2, r3, r4, r4_2, and accessory domains (AD) could be acquired (plain arrows) or lost (dashed arrows) during the evolution. Domain modification events of r4 into r4_2 domain are shown. Protein domain organizations are also displayed. $, means that the major AD is DUF6596.

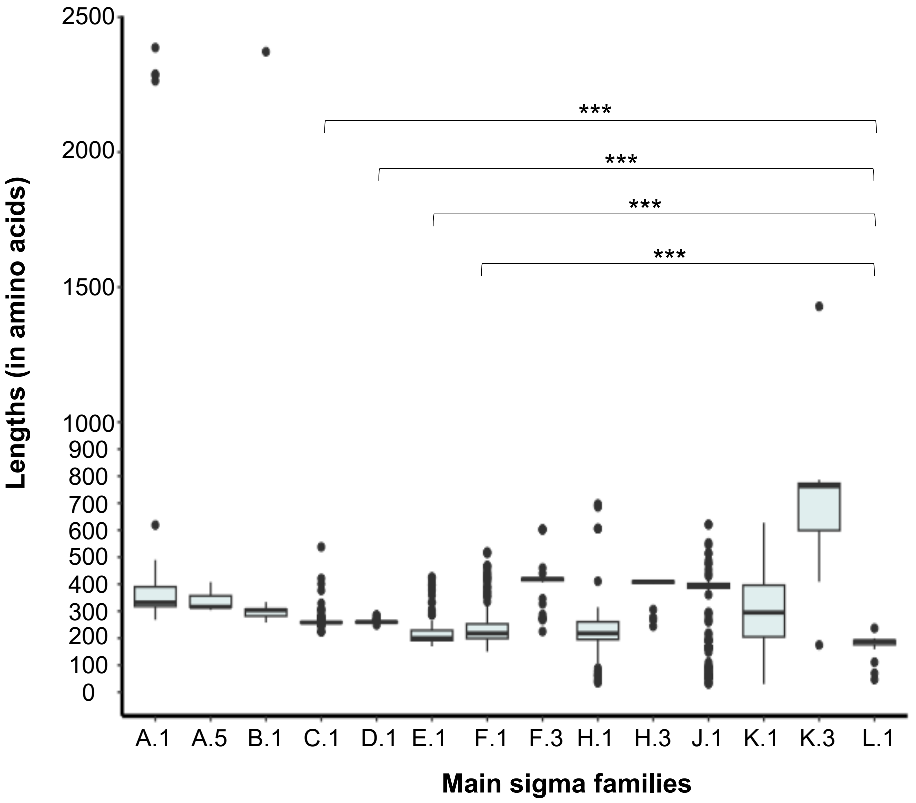

Fig. S7

Distribution of the sigma70 protein lengths in major families.

Statistical comparisons were performed between L.1 and [C.1, D.1, E.1, and F.1] families. *** is shown when the statistical significance (Wilcoxon-Mann-Whitney test, *p*-value) was below 0.001.

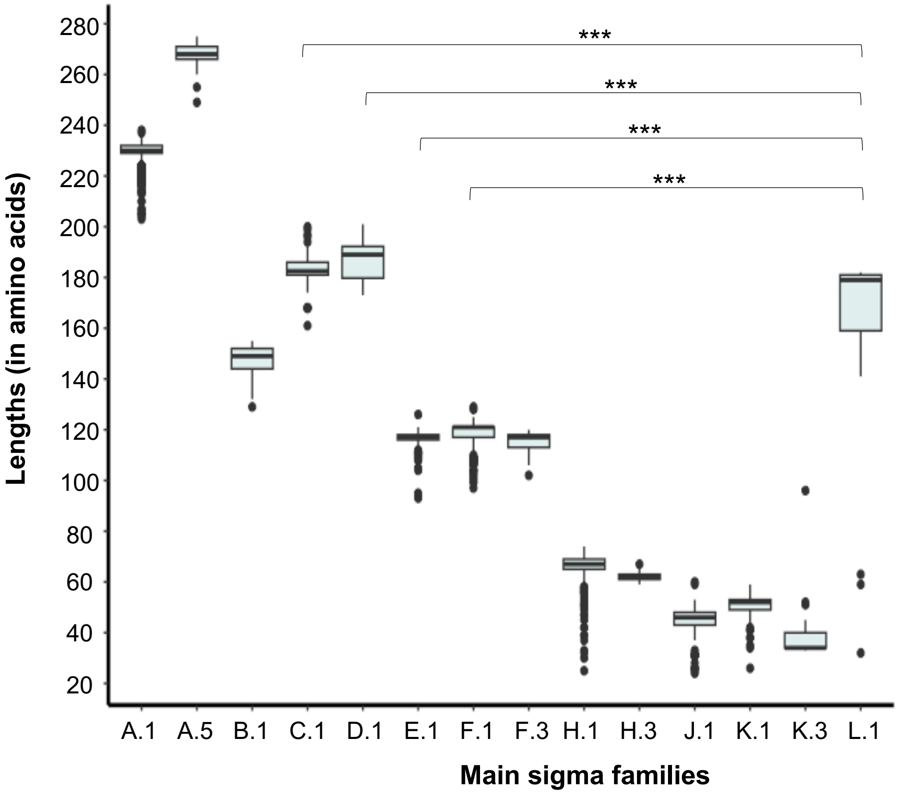

Fig. S8

Distribution of the total lengths of protein domain regions along the sigma70 proteins.

Statistical comparisons were performed between L.1 and [C.1, D.1, E.1, and F.1] families. *** is shown when the statistical significance (Wilcoxon-Mann-Whitney test, *p*-value) was below 0.001.

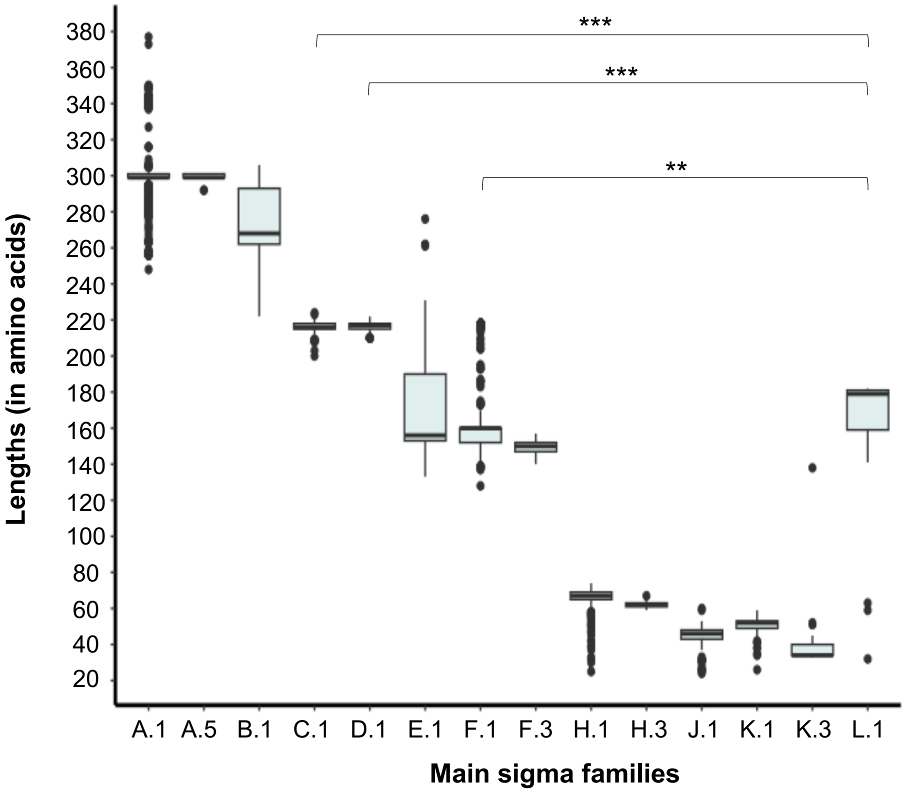

Fig. S9

Length distributions of protein regions covered by the sigma r domains.

Statistical comparisons were performed between L.1 and [C.1, D.1, E.1, and F.1] families. ** and *** are shown when the statistical significance (Wilcoxon-Mann-Whitney test, *p*-value) were below 0.01 or 0.001, respectively.

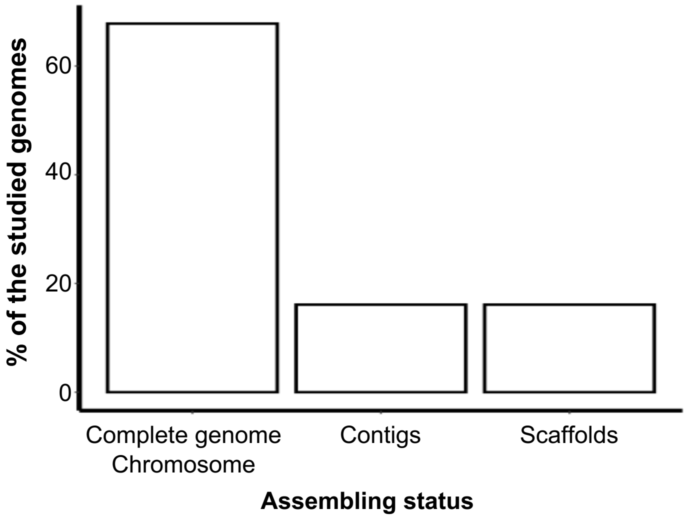

Fig. S10

Distribution of the studied genome assembly status.

Genome status information (Complete genome or in Chromosome, Contigs, and Scaffolds) were obtained from the NCBI database. The percentage is relative to the total number of genomes.

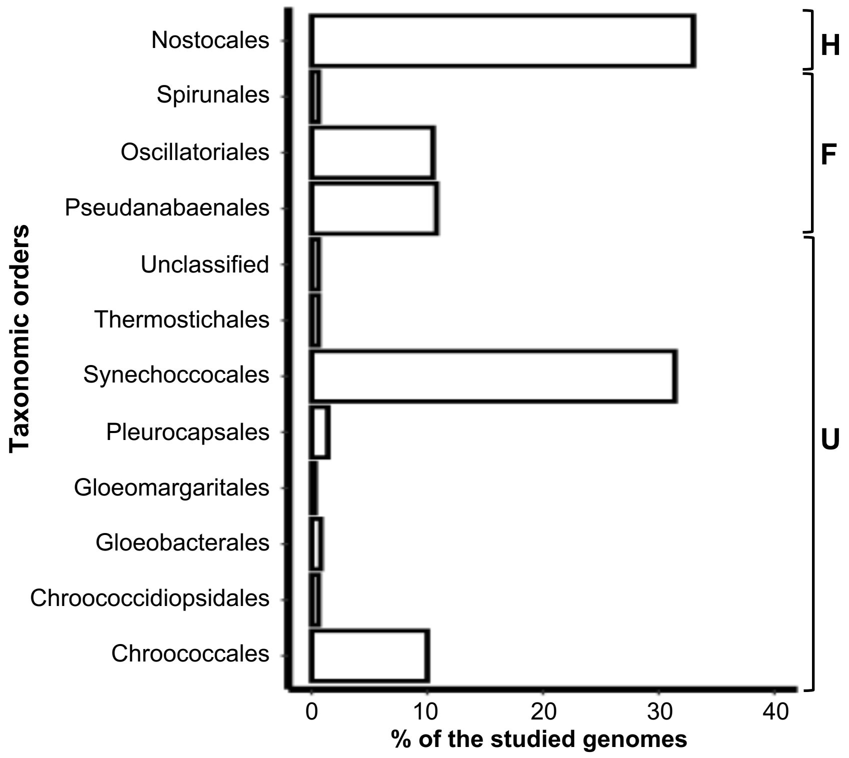

Fig. S11

Proportion of the studied genomes in cyanobacteria taxonomic orders.

The percentage is relative to the total number of genomes. Phenotypic traits of organisms are shown: U, unicellular; F, filamentous non-heterocyst-forming; and H, heterocyst-forming organisms.
